# A colour-tunable chiral AIEgen: reversible coordination, enantiomer discrimination and morphology visualization†
†Electronic supplementary information (ESI) available. CCDC 1472587. For ESI and crystallographic data in CIF or other electronic format see DOI: 10.1039/c6sc01614f


**DOI:** 10.1039/c6sc01614f

**Published:** 2016-06-02

**Authors:** Jesse Roose, Anakin Chun Sing Leung, Jia Wang, Qian Peng, Herman H.-Y. Sung, Ian Duncan Williams, Ben Zhong Tang

**Affiliations:** a HKUST-Shenzhen Research Institute , No. 9 Yuexing 1st RD , South Area , High-tech Park , Nanshan , Shenzhen 518057 , China . Email: tangbenz@ust.hk; b Department of Chemistry , Hong Kong Branch of Chinese National Engineering Research Center for Tissue Restoration & Reconstruction , Institute for Advanced Study , Institute of Molecular Functional Materials , Division of Biomedical Engineering , Division of Life Science and State Key Laboratory of Molecular Neuroscience , The Hong Kong University of Science & Technology , Clear Water Bay , Kowloon , Hong Kong , China; c Guangdong Innovative Research Team , SCUT-HKUST Joint Research Laboratory , State Key Laboratory of Luminescent Materials and Devices , South China University of Technology , Guangzhou 510640 , China; d Key Laboratory of Organic Solids , Institute of Chemistry , Chinese Academy of Sciences , Beijing 100190 , China

## Abstract

We present a conceptually new approach to synthesise a boron-containing Aggregation-Induced Emissive Luminogen (AIEgen) with a chiral chromophore. An intramolecular N–B coordinating bond results in a low-energy transition that renders the material red-emissive in a solid state. By competitive binding of nucleophiles to the boron atom, this bond is replaced in favour of an intermolecular coordinating bond, which results in a tremendous blue-shift in both the absorption and emission. A supportive DFT computation elucidates that a breakage of the intramolecular N–B coordinating bond causes a tremendous loss of conjugation in the LUMO, resulting in a larger energy gap. Owing to the fact that our scaffold is intrinsically chiral and Lewis-acidic, we demonstrate how our AIEgen discriminates between two pairs of enantiomers in a simple UV-vis measurement. Furthermore, the binding capabilities are exploited to stain polymer blends that comprised a non-coordinating and a Lewis-basic polymer. The red fluorescence that originates only from domains of the non-coordinating polymer is conveniently detected by a fluorescence microscope. Thus, compared to current analytical methods, we present a cheaper and faster methodology to study the micro-morphologies of certain polymer blends.

## Abstract 2

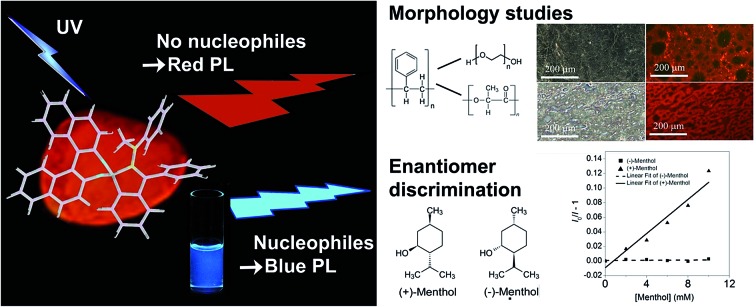
We present a chiral red-fluorescent AIEgen that reversibly coordinates nucleophiles with large changes in optical properties. This effect is used to discriminate enantiomers and analyse the micro-morphology of polymer blends.

## Introduction

Tetraphenylethene, silole, boron ketoiminate, and 9,10-distyrylanthracene derivatives are some prominent examples of fluorescent scaffolds that have been intensively studied during the last decade.^1^–^5^ What makes them so special compared to standard text book entries, such as fluorescein and rhodamines, is the fact that these scaffolds do not suffer from Aggregation-Caused Quenching (ACQ), but in contrast become emissive upon aggregation. In 2001, Tang and co-workers reported a pentaphenylsilole that was non-emissive in a solution but became emissive when aggregates were formed by the addition of a poor solvent; therefore, they introduced the term Aggregation-Induced Emission (AIE) to describe this phenomenon and AIEgen for the fluorophore showing such behaviour.^6^ Based on numerous fundamental studies, it turns out that the Restriction of Intramolecular Motion (RIM) is responsible for blocking the non-radiative pathway in the aggregated state. Thus, the excited AIEgen releases its excited-state energy to a significant extent by emitting a photon in the case where the non-radiative pathway is hindered. Since numerous applications relying on fluorescence require solid-state fluorescence, it is not surprising that AIEgens have received considerable attention. They often outperform their ACQ-suffering peers in modern opto-electronical applications such as organic light-emitting diodes (OLEDs) or organic field-effect transistors (OFETs). For staining cell organelles, AIEgens have proven superior to numerous commercial fluorophores.^5^,^7^

Rendering AIEgens chiral is of interest to furnish materials that possess Circularly-Polarised Luminescence (CPL) properties,^8^–^15^ the ability to discriminate between enantiomers,^16^–^23^ or to possibly bind selectively to chiral domains of bio-molecules for *in vivo* and *in vitro* staining. In this regard, a few examples appeared recently. However, all these examples have been based on tethering chiral functional groups to an otherwise achiral AIEgen. Such extrinsic approaches gave systems that exhibited pronounced chiroptical properties when allowed to aggregate under controlled conditions, which give rise to the occurrence of mostly helically chiral superstructures. While these systems generally benefit from an enhanced chirality and hence a tremendous amplification of their chiroptical properties, they lack the versatility that is required to operate in an individual fashion under various conditions, *i.e.* for fundamental studies of single-molecule CPL or binding to chiral domains of bio-molecules.^24^–^26^ Therefore, great interest has arisen in synthesising an intrinsically chiral AIEgen. Due to the fact that a structural key feature of most AIEgens lies in a phenyl ring-decorated core, numerous AIEgens feature propeller-like structures, which potentially can be rendered chiral. The intrinsically chiral AIEgens emerging from this structural manipulation would enjoy autonomy towards different environments and its chirality could be exploited without the necessity of forming self-assemblies.

The research on AIE-active systems has produced a myriad number of different AIEgens with a large variety of chemical and physical properties.^5^ Most of them respond to changes of the environment, *i.e.* viscosity and solubility, by means of changes in the fluorescence intensity. In addition, some donor–acceptor substituted AIEgens undergo colour-changes in the media with different polarities through Twisted Intramolecular Charge-Transfer (TICT). While such response to general changes in the environment is useful to obtain a broad picture, it often lacks the specificity and spatial resolution that are necessary to distinguish microenvironments in biological cells or in polymer blends. The latter are mostly composed of immiscible industrial polymers taking advantage of different mechanic and/or chemical properties arising from the respective polymers. These properties, however, tremendously depend on the microscopic composition of the blends.^27^ Hence, it is important to analyse the morphology, including parameters such as the size of the polymer domains. Commonly, these parameters have been obtained by Scanning Electron Microscopy (SEM) or Transmission Electron Microscopy (TEM) of films prepared from the respective blends. Occasionally, the sample has to be pre-treated with a tungsten complex to stain olefinic domains. In particular, for industrial applications, these techniques are time consuming and expensive. A simple optical measurement would therefore be desirable to lower the costs and accelerate the process. However, optical microscopes usually cannot provide enough information, due to a low penetration depth and lack of contrast between two different polymer domains.

In this study, we succeeded in furnishing a new boron-based AIEgen that possesses intrinsic chirality. (*R*)-1,1′-Bi-2-naphthol (BINOL) was used as an inexpensive, commercially available, and aromatic source to introduce chirality.^28^ We exploited the oxophilicity of boron to condense BINOL with a boronic acid-containing system under the same mild conditions that were recently utilized by James and co-workers.^29^,^30^ The resulting red-fluorescent, intrinsically chiral AIEgen revealed another interesting feature: the boron atom was enveloped in an aza-heterocyclic system^31^,^32^ due to the fact that a neighbouring hydrazone formed a coordinating N–B bond as evidenced by X-ray crystal analysis. A supporting DFT calculation provided insight into the electronic structure, most importantly that the LUMO enjoyed a relatively low energy through this coordinating bond. In solutions containing a Lewis-base, the boron atom underwent competitive binding, resulting in replacement of the intramolecular N–B bond. This caused a tremendous blue-shift for both the absorption spectrum and the photoluminescence spectrum, which we utilized to discriminate between Lewis-basic pairs of enantiomers and to study the micro-morphologies of polymer blends that were composed of a non-coordinating and a Lewis-basic polymer.

## Results & discussion

### Synthesis

**Scheme 1:**
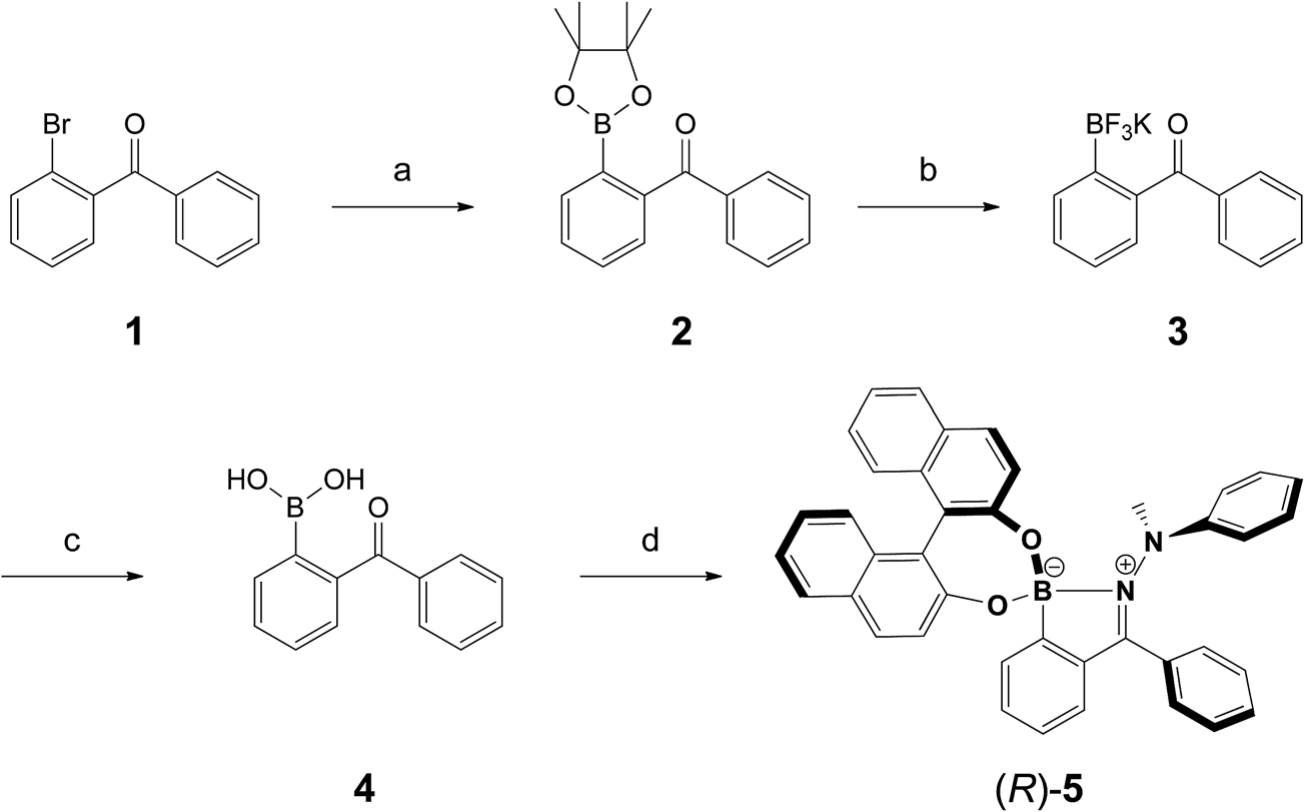
Synthesis of (*R*)-**5**. (a) B_2_pin_2_, [PdCl_2_(dppf)], KOAc, THF, 65 °C, 18 h, 84%; (b) KHF_2(aq)_, 1 : 1 MeOH/THF, 22 °C, 15 min, 92%; (c) LiOH·H_2_O, 2 : 1 MeCN/H_2_O, 22 °C, 24 h, 100%; (d) (1) 1-methyl-1-phenylhydrazine, MgSO_4_, CHCl_3_, 22 °C, 30 min; (2) (*R*)-BINOL, 70 °C, 2 d, 73%.

Commercially available 2-bromobenzophenone (**1**) was treated under Suzuki–Miyaura conditions^33^ to obtain **2** in good yields (Scheme 1). Applying conditions reported by Yuen *et al.*, boric ester **2** was converted into its corresponding trifluoroborate **3**.^34^ In order to obtain the free boronic acid **4**, trifluoroborate **3** was hydrolysed with lithium hydroxide. This two-step conversion of the pinacol ester **2** into the corresponding boronic acid **4** has proven superior in terms of yield and ease of purification compared to the standard single-step procedure in which sodium periodate is utilized to oxidatively cleave pinacol. Inspired by a three-component reaction reported by James and co-workers,^35^ boronic acid **4** was converted into (*R*)-**5***via* a two-step, one-pot sequence, involving the formation of an intermediate hydrazone from 1-methyl-1-phenylhydrazine. A test experiment using the same conditions in deuterated chloroform has previously shown completion of the hydrazone formation in less than 30 min. By the subsequent addition of (*R*)-BINOL and heating for two days at 70 °C in a sealed pressure tube, the target compound (*R*)-**5** was furnished as an orange-red coloured powder in 56% yield over four steps. The structure of (*R*)-**5** was unambiguously assigned by X-ray crystal analysis (see next paragraph). The rotation around the N–N-bond leads to a 1 : 2 mixture of diastereoisomers, as evidenced by ^1^H NMR (Fig. S8†). The X-ray crystal analysis, however, only showed one diastereoisomer, presumably because the thermodynamically favoured structure was regenerated in equilibrium.

### X-ray crystal structure analysis

**Fig. 1:**
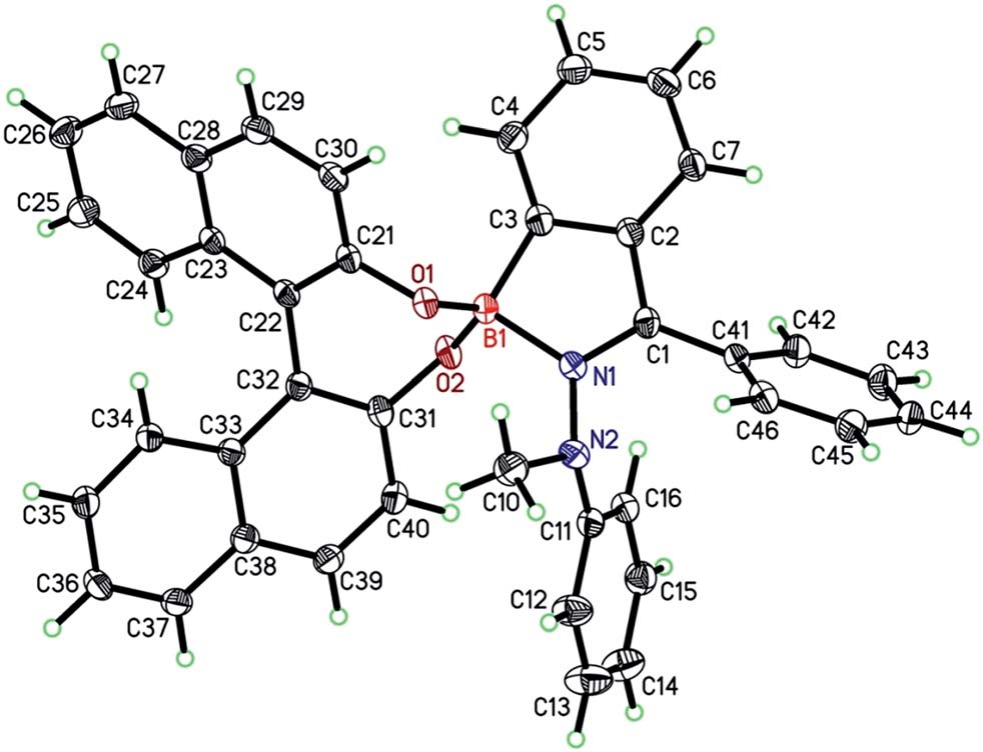
ORTEP plot of (*R*)-**5**; orthorhombic, space group *P*2_1_2_1_2_1_. Arbitrary numbering, atomic displacement parameters obtained at 100 K are drawn at 40% probability level.

The ORTEP plot of (*R*)-**5** is shown in Fig. 1, proving unambiguously the proposed structure of (*R*)-**5**. The molecular structure exhibited two noteworthy features: first, the axial chirality of the BINOL-substituent (*θ*_(C21–C22–C32–C31)_ = 49.6°) was transferred to the 1-methyl-1-phenylhydrazone moiety (*θ*_(C1–N1–N2–C11)_ = 81.4°) and even further to the pendant phenyl ring on C1 (*θ*_(C2–C1–C41–C42)_ = 51.6°). Second, the boron–nitrogen bond length *d*_(B1–N1)_ amounts to 1.66 Å and hence constitutes a very long bond compared to others between two second-period elements. Usually, the B–N bond-lengths in tri-coordinated boron-compounds average at around 1.4 Å.^36^ Previously, longer bond-lengths have also been found for some tetra-coordinated boron-species. Höpfl reported that the B–N length in tetra-coordinated species can vary from 1.57 to 2.91 Å.^37^ In line with this report, Höpfl and co-workers found a B–N distance of 1.61 Å for a salen-ligated dinuclear boron complex.^38^ For 4,4-difluoro-BODIPY scaffolds, even shorter B–N bond lengths of 1.55 Å have been reported.^39^ Quinoxaline-derived boron-ketoiminates possess B–N distances of around 1.59 Å.^40^ Due to the elongation in (*R*)-**5**, the bond is weakened and thus expected to be liable to breakage in the presence of a competing Lewis-base.

### Photo-physical properties

**Fig. 2:**
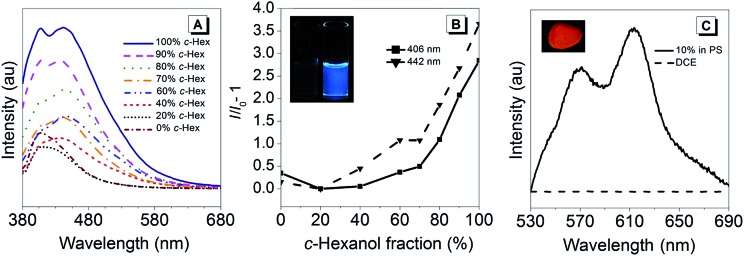
Fluorescence spectra (A) of (*R*)-**5** in different THF–*c*-hexanol mixtures (10 μM). (*R*)-**5** is well soluble in both solvents, *c*-hexanol hinders molecular motion due to its high viscosity (57.5 cP) as opposed to that of THF (0.46 cP). Excitation at 360 nm. (B) Plot of the fluorescence maxima at 406 and 442 nm as a function of the *c*-hexanol fraction. The inset shows THF (left) and *c*-hexanol (right) solutions (both 10 μM) under a hand-held UV-lamp (364 nm). (C) PL spectrum of (*R*)-**5** (10 wt%) in polystyrene (PS). Excitation at 480 nm. The inset shows the film under excitation with a hand-held UV-lamp (364 nm).

In order to investigate the AIE-properties of (*R*)-**5**, the common methods based on measuring the photoluminescence intensities in solvent mixtures that cover the range from excellent to poor solubility were not applicable. The reason relates to the observation that (*R*)-**5** suffered from slow hydrolysis in THF-water mixtures. In addition, the solubility in common poor solvents such as alkanes was found to be very high to form aggregates at low concentrations, which are generally necessary for measuring photoluminescence. However, it turned out that THF–*cyclo*-hexanol mixtures provided a system suitable to demonstrate the AIE-properties of (*R*)-**5**. Although (*R*)-**5** is soluble in *cyclo*-hexanol, it is its comparatively high viscosity of 57.5 cP – the viscosity of THF is 0.46 cP – that leads to an attenuation of the intramolecular motions (cP = centipoise, a standard unit of measurement for viscosity).^41^ The photoluminescence spectra for different THF–*cyclo*-hexanol mixtures are depicted in Fig. 2A. To our surprise, compound (*R*)-**5** underwent a tremendous blue-shift. The initially orange-red powder gave light-yellow solutions, which exhibited blue fluorescence in THF–*cyclo*-hexanol and featured two emission peaks with maxima at 406 and 442 nm. A solution of (*R*)-**5** in pure THF did not show any fluorescence discernible by the naked eye, although a weak fluorescence was detected in the photoluminescence measurement. However, increasing the viscosity by adding *cyclo*-hexanol lead to visible blue fluorescence (Fig. 2B, inset), which increased by a factor of approximately four. This enhancement is typical for AIEgens in media with different viscosities.^42^,^43^ In absolute terms, (*R*)-**5** exhibited a quantum yield *Φ*_em_ of 4% in the solid-state and *Φ*_em_ = 0.1% in solution.

The tremendous blue-shift prompted us to compare the emission in the solid state and in a solution using a solvent that did not lead to a colour change. As such, 1,2-dichloroethane (DCE) was chosen. For the solid-state measurement, a dispersion of (*R*)-**5** in polystyrene (PS, 10 wt%) was prepared by mixing PS with (*R*)-**5** in a toluene solution that was subsequently drop-casted on a glass plate to give a red-fluorescent film. While the DCE solution did not exhibit any fluorescence, the film showed intense red fluorescence having two distinct bands with maxima at 568 and 615 nm (Fig. 2C). Such a bathochromic shift was also reflected in the absorption spectra.

**Fig. 3:**
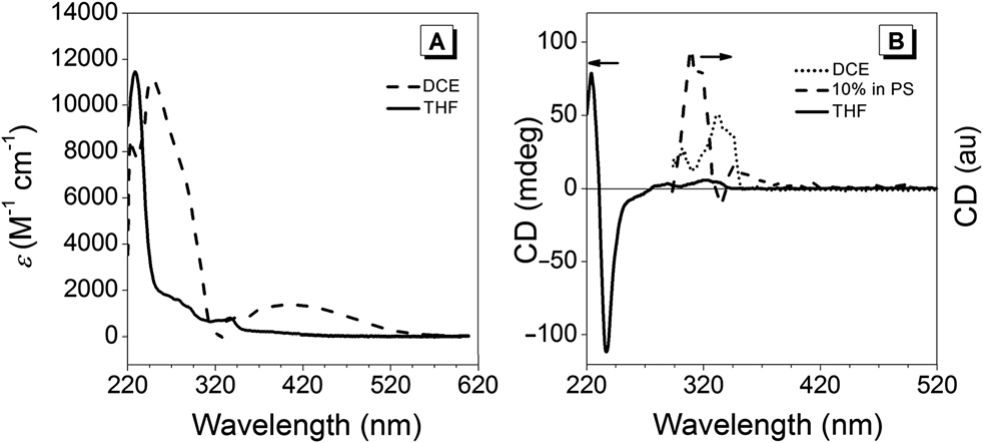
Absorption spectra (A) in 1,2-dichloroethane (DCE; dashed trace) and THF (solid line); *c* = 10^–5^ M. CD spectra (B) in THF (solid line, *c* = 10^–5^ M), as a film in PS (dashed line, 10%) and in DCE (dotted trace; *c* = 10^–5^ M).

Fig. 3A illustrates the UV-vis spectra of (*R*)-**5** in 1,2-dichloroethane (DCE; solid trace) as well as in THF (dashed trace). Below 300 nm, a bathochromic shift of about 20 nm was observed for the DCE solution compared to the spectrum recorded in THF, whereas the band at 333 nm did not undergo a shift within the margin of error. Interestingly, a broad band with a maximum at 403 nm extending into the blue-green region above 500 nm, which was observed when measured in DCE, was absent in the THF solution. This effect was also observed by the naked eye: (*R*)-**5** solutions in solvents that have Lewis-basic groups (alcohols, ethers and ketones) were yellow as opposed to those in non-coordinating solvents such as hydrocarbons or chlorinated solvents, which featured an intense orange-red colour similar to the material in the solid state. Considering the data obtained from the X-ray crystal analysis, a competitive coordination of Lewis-basic solvents with boron is very likely to occur, resulting in B–N bond cleavage (for further details, see the previous paragraph). Such competitive binding would alternate the chromophore, leading to a hypsochromic shift and the disappearance of the broad band at 365 nm. The CD traces in Fig. 3B show that the chirality in THF solutions only arose from the BINOL moiety, whereas Cotton bands at longer wavelengths were observed in a non-coordinating DCE solution or in a solid polystyrene matrix. This observation further implied that breakage of the B–N bond occurs in nucleophilic solvents since the open form would enjoy a higher degree of freedom that could lead to the loss of the chirality transfer from the BINOL to the hydrazone scaffold.

**Fig. 4:**
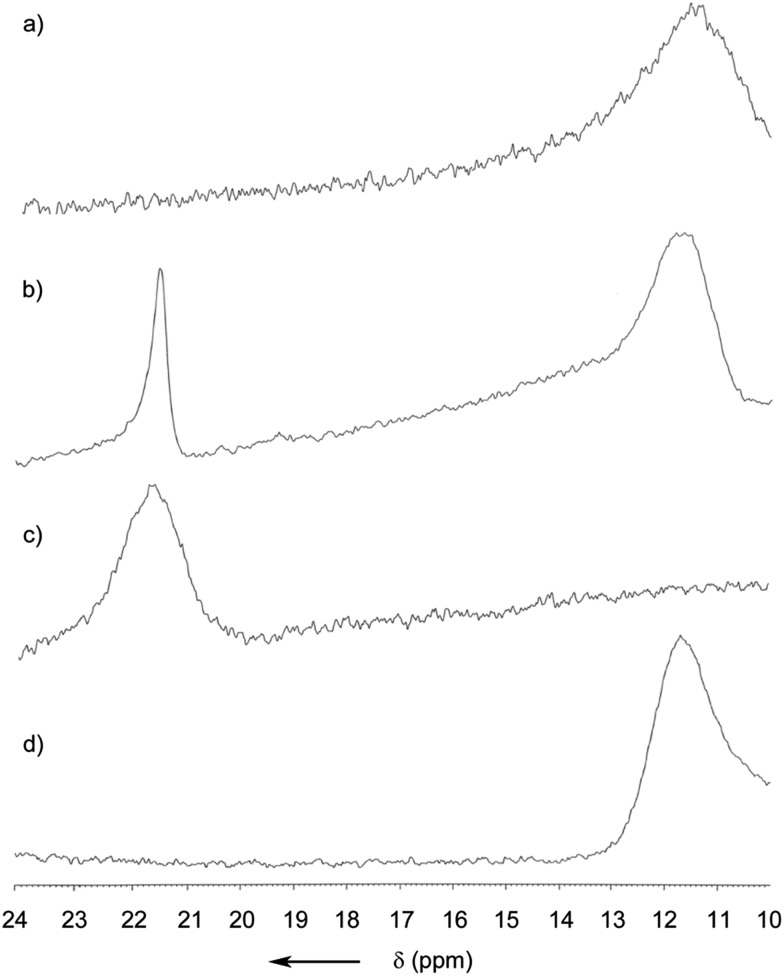
^11^B NMR spectra of (*R*)-**5** in CDCl_3_ (a), with 10 eq. of THF (b), with an excess of THF (c), and (d) after drying and re-dissolving the sample in CDCl_3_.

For obtaining a better understanding of the role of the B–N bond, a DFT calculation was performed at the B3LYP/6-31G(d) level (Fig. S12†). It was found that the electron density of the HOMO was mostly located at the electron rich BINOL-moiety and that the LUMO is predominantly situated around the heterocycle, the annulated benzene ring and the phenyl residue at C1 (see Fig. 1). Since part of the LUMO stretches in a π-fashion across the bonds connecting C3–B1–N1 (see Fig. 1), it appears reasonable to assume that a break of the B1–N1 bond would lead to a significant increase in the LUMO energy. Indeed, on calculating the open or non-coordinated isomer using the same parameters, we found a LUMO energy that is significantly higher compared to the closed form (Fig. S13†) with the HOMO being located at the electron rich hydrazone part and the LUMO at the comparatively less electron rich BINOL moiety. We found HOMO–LUMO energy gaps of 2.65 and 3.53 eV for the closed and open form, respectively. Hence, the coordination of a Lewis-base to the boron atom can induce a significant blue-shift both in the absorption and also the emission of (*R*)-**5**. Our hypothesis was further confirmed by a careful analysis of the MALDI-ToF mass spectrum (Fig. S11†). Not only did we observe the peak corresponding to the molecule cation [(*R*)-**5**]^+^ but also the peaks corresponding to adducts with nucleophiles, such as the matrix, or with less intensity, phthalates that are commonly used as plastic softeners.^44^ Furthermore, boron-11 NMR analysis revealed a distinct new peak ranging from 21 to 23 ppm after the addition of THF to the CDCl_3_ solution (Fig. 4). At an excess of THF, the original peak between 10 and 13 ppm disappeared entirely. However, it was regenerated after the sample was dried in vacuum (100 mbar, 40 °C) and re-dissolved in CDCl_3_ indicating reversibility. Such a chemical shift for (*R*)-**5** in the non-coordinated state was expected according to previous reports.^45^ The downfield shift of around 10 ppm can be explained by the replacement of the B–N coordination by a weaker R_2_O → B(OR′)_2_Ar coordinating bond. In addition, the reversibility of the coordination was also demonstrated by the restoration of the optical properties after removing THF.

### Enantioselective sensing

**Fig. 5:**
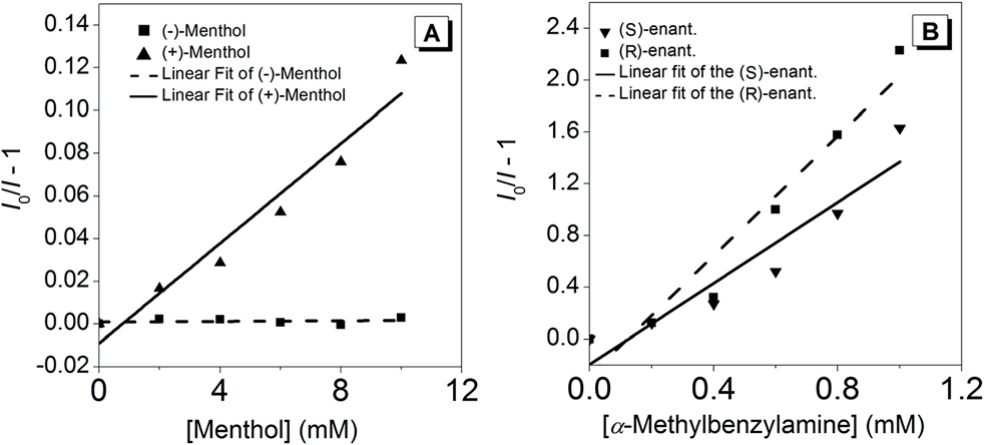
(A) Stern–Volmer-plots with *K*((–)-menthol) = 7 × 10^–5^ M^–1^ (*y* = 7 × 10^–5^*x* + 0.0008; *R*^2^ = 0.0401) and *K*((+)-menthol) = 0.012 M^–1^ (*y* = 0.0117*x* – 0.009; *R*^2^ = 0.9447). (B) Stern–Volmer-plots with *K*((*R*)-α-methylbenzylamine) = 2.31 M^–1^ (*y* = 2.3103*x* – 0.2802; *R*^2^ = 0.9407) and *K*((*S*)-α-methylbenzylamine) = 1.57 M^–1^ (*y* = 1.565*x* – 0.1966; *R*^2^ = 0.9008); as determined from the slope of the linear fit line.

Since (*R*)-**5** is a chiral Lewis-acid undergoing reversible coordination to Lewis-bases, we investigated whether two enantiomeric alcohols would bind with different binding constants depending on their chirality. For their relevance and availability, (+)- and (–)-menthol were tested. As mentioned above, the coordination of a Lewis-base to the boron atom leads to a quenching of the broad absorption band at *λ*_max_ = 403 nm. Hence, the UV-vis spectra at different menthol concentrations, ranging from 0 to 10 mM, were recorded (Fig. S14†) and the data were analysed through a Stern–Volmer-Plot (Fig. 5A) by plotting the respective intensities at 400 nm. While the naturally occurring (–)-menthol only showed a binding constant of 7 × 10^–5^ M^–1^, the artificial (+)-enantiomer exhibited a 150-fold increase in binding (*K* = 0.012 M^–1^).

To extend the scope, the binding properties between a chiral amine, namely, α-methylbenzylamine, and (*R*)-**5** were investigated. Due to the stronger Lewis-basicity of amines, the concentration of the enantiomers was chosen one order of magnitude lower compared to menthol. The Stern–Volmer-plots, shown in Fig. 5B, revealed that both enantiomers bound to (*R*)-**5**. However, (*R*)-α-methylbenzylamine featured a binding constant, *K*, 1.5 times higher than its antipode. The reason for the lower ratio between the binding constants as opposed to those of (+)- and (–)-menthol can be found in two facts: (i) the amino group is sterically less hindered causing the corresponding diastereoisomeric complexes with (*R*)-**5** to be thermodynamically more equal; (ii) a stronger binding is expected due to the higher Lewis-basicity of the amino group.

### Staining polymer blends

**Fig. 6:**
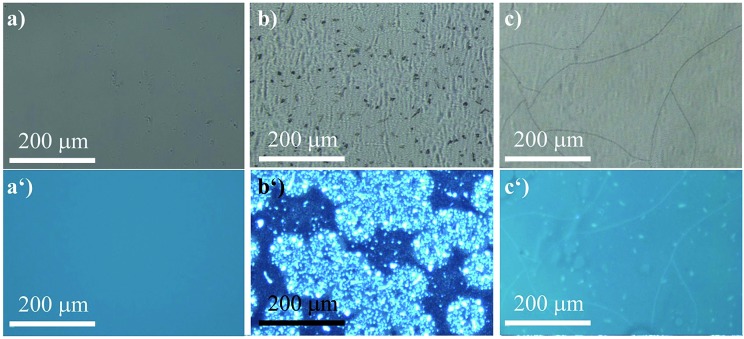
Micrographs of the TPE-stained (2 wt%) films composed of pure PS (a) bright-field and (a′) *λ*_ex_ = 330–385 nm, filter > 390 nm), of pure PEG (b) bright-field and (b′) *λ*_ex_ = 330–385 nm, filter > 390 nm), and PS–PEG 75 : 25 (c) bright-field and (c′) *λ*_ex_ = 330–385 nm, filter > 390 nm).

The ability of (*R*)-**5** to reversibly undergo coordination to Lewis-basic groups was further exploited to stain polymer blends that were composed of a non-coordinating polymer and a polymer containing Lewis-basic groups. Common AIEgens such as TPE fail to differentiate between mixtures of rigid polymers.^46^ For optically analysing the polymer blends, the polymers were dissolved in toluene and stained with AIEgen (2 wt%). Subsequently, the resulting solutions were spin-coated onto glass substrates. Further details can be found in the experimental section. Indeed, we demonstrated that TPE cannot differentiate between a blend composed of polystyrene (PS) and microcrystalline polyethylene glycol (PEG) regardless of their huge structural differences (Fig. 6). It was expected that the amorphous PS would allow for more structural flexibility compared to PEG, which develops spherulite structures, thus restricting the intramolecular motion to a larger extent. This assumption relates to the fact that TPE can only yield different fluorescence intensities as a function of the viscosity or rigidity of the surrounding medium. However, the sensitivity of TPE to differentiate between PS and PEG was found to be not high enough for visualisation. In addition, TPE offers no colour tunability. Thus, both the PS and PEG films showed blue fluorescence, as illustrated in Fig. 6a, a′ and b, b′. Notably, Fig. 6b′ shows the microcrystalline phases of the PEG that appear in spherulites. As a consequence, the investigated PS–PEG blend (75 : 25 wt%) did not exhibit any differences that would allow for analysis of the micro-morphologies (Fig. 6c and c′). Earlier experiments in which PEGs with different molecular weights were employed suggested that the degree of crystallinity did not influence the emission properties to an extent that could be used to unambiguously differentiate between the two polymer phases.

**Fig. 7:**
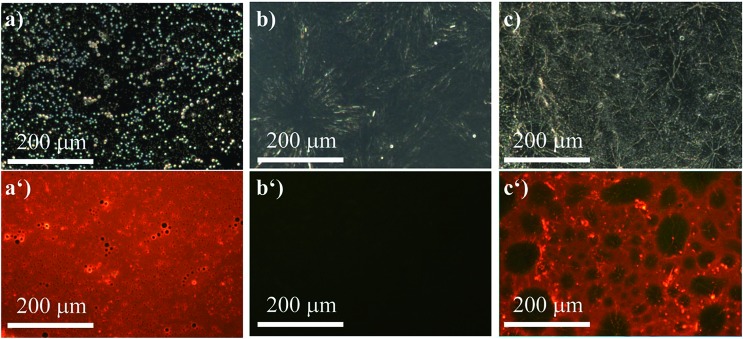
Micrographs of the (*R*)-**5**-stained (2 wt%) films composed of pure PS (a) bright-field and (a′) *λ*_ex_ = 400–440 nm; filter > 455 nm, of pure PEG (b) bright-field and (b′) *λ*_ex_ = 400–440 nm; filter > 455 nm), and PS–PEG 75 : 25 (c) bright-field and (c′) *λ*_ex_ = 400–440 nm; filter > 455 nm.

However, compound (*R*)-**5** provides a versatile tool to overcome these obstacles in case one of the polymers contains nucleophiles, *i.e.* oxygen or nitrogen atoms. We first investigated the same polymer blend composed of PS and PEG. Owing to the ability of (*R*)-**5** to dynamically and reversibly coordinate to the Lewis-basic oxygen atoms in the PEG-chains, which is accompanied by fluorescence quenching, only the PS-phases remained red-fluorescent as evidenced by the fact that the pure PS-film (Fig. 7a and a′) exhibited a uniform red fluorescence. The film composed of PEG (Fig. 7b and b′) by contrast did not show any signal at *λ*_ex_ = 400–440 nm (filter > 455 nm). In Fig. 7c and c′, the benefit of our AIEgen becomes obvious. While the bright-field image (Fig. 7c) suggested a homogeneous composition, the fluorescence micrograph clearly showed the different spherical domains of PS and PEG. Therefore, not only were we able to show the micro-morphology but we could also unambiguously assign the different domains to the respective polymers; here the red-fluorescent phase correlated to PS and the non-fluorescent phase to PEG. A quick screen of different ratios of our dye-polymer composition revealed an optimal content of 2 wt%. Lower contents sometimes did not provide clear micrographs.

**Fig. 8:**
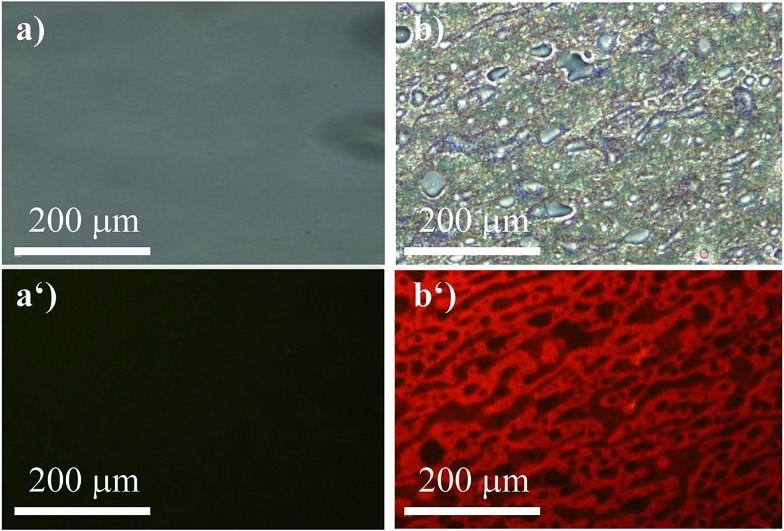
Micrographs of the (*R*)-**5**-stained (2 wt%) films composed of pure PLA (a) bright-field and (a′) *λ*_ex_ = 400–440 nm; filter > 455 nm and PS–PLA 50 : 50 (b) bright-field and (b′) *λ*_ex_ = 400–440 nm; filter > 455 nm.

To widen our scope, we chose a polymer that recently received lots of interest not so much for its mechanical properties but for the fact that it is biodegradable, namely, polylactic acid (PLA).^47^ Due to its poor mechanical performance, it is commonly blended with another polymer to achieve a material with reasonable mechanical properties. Hence, we formed a blend with PS (50 : 50) that was stained with (*R*)-**5** (Fig. 8). Similar to the previous example, the red fluorescence of (*R*)-**5** was entirely quenched in pure PLA (Fig. 8a′). That fact was exploited to study the micro-morphology of the blend (Fig. 8b and b′). Although the phase separation between PS and PLA was partly visible in the bright-field image, it lacked the depth and the accuracy to provide for a meaningful analysis. Under excitation, however, the morphology became clearly visible (Fig. 8b′) and revealed a co-continuous structure.

Thus, we demonstrated a feasible tool to study micro-morphologies with relatively cheap and fast optical microscopy owing to the fact that (*R*)-**5** selectively stained non-coordinating polymers.

## Conclusions

We introduced a conceptually new chiral boron-based AIEgen, which was furnished in good yields, by applying a short synthetic sequence that provides possibilities to facilely introduce additional functional groups. The molecular design of the AIEgen was novel and the chromophore itself was chiral. Interestingly, interactions of nucleophiles with the boron atom lead to a reversible breakage of an intramolecular N–B coordinating bond. Since the intramolecular N–B bond gave rise to a low-lying LUMO, a pronounced blue-shift was observed in the presence of Lewis-bases. This finding and the fact that the presented structure is chiral was exploited to discriminate between pairs of enantiomers that contained common functional groups, such as an alcohol or an amino group. The detection method was based on the partial quenching of the long-wavelength absorption band, which peaks at 403 nm; thus, the enantiomer discrimination only required a simple set-up to obtain the absorption spectra. In the case of menthol, it was found that only the (+)-enantiomer significantly interacts with our AIEgen in a concentration range of 1–10 mM. Due to less steric hindrance and stronger Lewis-basicity, both enantiomers of α-methylbenzylamine coordinated to our dye. However, (*R*)-α-methylbenzylamine formed a thermodynamically more favored complex, quenching the absorption in a linear fashion at low concentrations between 0.1 and 1 mM. Furthermore, our dye allowed us to venture into the analysis of the microscopic morphology of polymer blends. Again, we found that only a simple experimental set-up, namely, a common fluorescence microscope, was required to receive unambiguous data, which showed the microscopic composition between a non-coordinating and a Lewis-basic polymer. Since such studies, which are of great importance for the polymer-manufacturing industry, are currently conducted using time- and cost-intensive methods such as Scanning Electron Microscopy (SEM) and Transmission Electron Microscopy (TEM), our approach provides a remunerative alternative. In addition, our concept might inspire future work on modifications of the presented AIEgen that show CPL without the necessity of forming complex supramolecular structures.

### Experimental part

### Materials and general methods

Solvents and commercially available reagents were purchased from Sigma-Aldrich, J&K Chemicals, TCI, Fluka, and Labscan and used without further purification. The polymers were purchased from Sigma-Aldrich (PS; *M*_w_ = 280 000, *T*_g_ = 100 °C, *T*_m_ = 200 °C, amorphous and PEG; *M*_w_ = 3,600, *T*_g_ = –60 °C, *T*_m_ = 55–60 °C, degree of crystallinity >90%) and Chuang Hui Plast LTD (PLA; *M*_w_ = 60 000, *T*_g_ = 60–70 °C, *T*_m_ = 170–180 °C, degree of crystallinity approx. 37%). All reactions were conducted under an atmosphere of nitrogen unless otherwise stated. Solvents were purchased in p.a. quality. THF was dried with sodium/benzophenone and freshly distilled prior to its use. All products were dried under high vacuum (10^–2^ Torr) before analytical characterization. Thin-layer chromatography (TLC) was conducted on aluminium plates coated with SiO_2_-60 UV_254_ from Merck. Visualization was achieved by UV light at 254 nm. Flash column chromatography (FC) was performed using SiO_2_-60 (230–400 mesh, 0.040–0.063 mm) from Grace Davison Discovery Sciences with a head pressure of 0–0.4 bar. NMR spectra (^1^H, ^13^C, ^11^B, ^19^F) were obtained on a Bruker AV-400 at 23 °C using the solvent peak as an internal reference. Coupling constants (*J*) are given in Hz. The resonance multiplicity is described as s (singlet), d (doublet), t (triplet), q (quartet), sext. (sextet), sept. (septet), and m (multiplet). Broad signals are described as br. (broad). Mass spectra (MS) were obtained on a GCT premier CA B048 mass spectrometer using the MALDI-ToF mode with *trans*-2-[3-(4-*tert*-butylphenyl)-2-methyl-2-propenylidene]malononitrile (DCTB) as the matrix. UV spectra were acquired on a Milton Ray Spectronic 3000 Array spectrophotometer and photoluminescence spectra were acquired on a PerkinElmer LS 55 spectrophotometer. Electronic circular dichroism spectra were obtained on a Bio-logic MOS-450 spectropolarimeter. Fluorescence micrographs were taken on FL microscope (BX41 Microscope) using a combination of excitation and emission filters for each dye. The fluorescence images were captured using a computer-controlled SPOT RT SE 18 Mono charge-coupled device (CCD) camera.

### Phenyl[2-(4,4,5,5-tetramethyl-1,3,2-dioxaborolan-2-yl)phenyl]methanone^48^ (**2**)

A degassed (N_2_, 30 min) solution of 2-bromobenzophenone (**1**) (5.15 g, 19.7 mmol), bis(pinacolato)diboron (6 g, 23.7 mmol), and KOAc (5.7 g, 58 mmol) in dry THF (125 mL) was treated with [Pd(dppf)Cl_2_] (721 mg, 0.99 mmol) and heated at 65 °C for 18 h. The mixture was diluted with Et_2_O (200 mL), washed with water (3 × 50 mL), brine (1 × 50 mL), dried over anhydrous MgSO_4_, and evaporated *in vacuo* to give an oily residue. Column chromatography (SiO_2_; *n*-hexane/EtOAc 95 : 5) afforded the pure compound as a white solid (5.1 g, 84%).

*R*_f_ = 0.70 (SiO_2_; *n*-hexane/EtOAc 8 : 2); analytical data conform with those reported previously.^48^

### Potassium benzophenone-2-yltrifluoroborate (**3**)

A stirred solution of **2** (900 mg, 2.9 mmol) in MeOH/THF (1 : 1, 20 mL) was treated with an aqueous KHF_2_ solution (4.5 M, 4 mL, 16.4 mmol) at 22 °C for 15 min resulting in a cloudy mixture, which was subsequently concentrated *in vacuo*. The residue was dissolved in hot acetone, filtered, and evaporated *in vacuo* affording crude **3**. Subsequent recrystallization from acetone/Et_2_O gave colourless crystals (769 mg, 92%).

^1^H NMR (400 MHz, acetone-*d*_6_) *δ* = 7.82–7.70 (m, 3H), 7.57 (dd, *J* = 6.8, 1.5 Hz, 1H), 7.48–7.39 (m, 2H), 7.32 (dd, *J* = 7.0, 1.5 Hz, 1H), 7.20 (dd, *J* = 7.0, 1.5 Hz, 1H), 7.06–7.00 ppm (m, 1H); ^13^C NMR (101 MHz, acetone-*d*_6_) *δ* = 202.05, 143.00, 138.52, 133.25, 132.32, 130.25, 127.88, 125.61, 124.69 ppm; ^11^B NMR (128 MHz, acetone-*d*_6_) *δ* = 3.31 ppm (br. q); ^19^F NMR (376 MHz, acetone-*d*_6_) *δ* = 138.48 ppm (br. d).

### Benzophenon-2-ylboronic acid (**4**)

A solution of **3** (374 mg, 1.3 mmol) in acetonitrile/water (2 : 1, 15 mL) was treated with LiOH·H_2_O (191 mg, 4.5 mmol) and stirred for 24 h at 22 °C. The mixture was acidified with conc. aqueous NH_4_Cl (8 mL) and hydrochloric acid (1 M, 2 mL), extracted with EtOAc (3 × 10 mL), dried over anhydrous MgSO_4_, and evaporated *in vacuo* to afford boronic acid **4** as a white solid (293 mg, 100%).

^1^H NMR (400 MHz, acetone-*d*_6_) *δ* = 7.80–7.68 (m, 3H), 7.63 (t, *J* = 6.8 Hz, 1H), 7.61–7.55 (m, 2H), 7.55–7.47 (m, 3H), 2.83 ppm (s, 2H); ^13^C NMR (101 MHz, acetone-*d*_6_) *δ* = 196.56, 143.85, 137.67, 133.22, 131.91, 129.30, 129.25, 129.07, 127.72, 127.67 ppm; ^11^B NMR (128 MHz, acetone-*d*_6_) *δ* = 31.10 ppm (br.).

### (*R*)-2-{[2-(Dinaphtho[2,1-*d*:1′,2′-*f*][1,3,2]dioxaborepin-4-yl)phenyl](phenyl)methylene}-1-methyl-1-phenylhydrazine ((*R*)-**5**)

A pressure tube was charged with boronic acid **4** (194 mg, 0.86 mmol), anhydrous MgSO_4_ (500 mg, 4.2 mmol), CHCl_3_ (5 mL), and 1-methyl-1-phenyl-hydrazine (100 μL, 0.86 mmol). After stirring this mixture for 30 min at 22 °C, (*R*)-BINOL (246 mg, 0.86 mmol) was added and the mixture heated to 70 °C for 2 d. The mixture was filtered, the filtrate evaporated *in vacuo*, and filtered again over alumina (10 g) with *n*-hexane (20 mL) and Et_2_O (10 mL) to give a 2 : 1 diastereoisomeric mixture of (*R*)-**5** as an orange-red powder (364 mg, 73%).

^1^H NMR (400 MHz, CDCl_3_) *δ* = 7.98 (d, *J* = 8.5 Hz, 2H), 7.88 (d, *J* = 8.5 Hz, 2H), 7.81–7.62 (m, 2H), 7.60–7.41 (m, 5H), 7.41–7.33 (m, 5H), 7.33–7.27 (m, 4H), 7.18–7.12 (m, 3H), 6.88 (d, *J* = 8.5 Hz, 2H), 3.37 and 3.31 ppm (s, 3H); ^13^C NMR (101 MHz, CDCl_3_) *δ* = 152.12, 148.02, 133.36, 132.77, 131.86, 131.00, 130.80, 130.01, 129.93, 128.98, 128.89, 128.82, 128.60, 128.30, 128.05, 127.95, 127.78 (br.), 127.62, 127.38, 127.22, 126.85, 126.23, 123.57, 123.41, 122.00, 121.93, 117.13, 110.20, 41.49 ppm; ^11^B NMR (128 MHz, CDCl_3_) *δ* = 11.09 ppm (br.); HRMS (MALDI): calcd for C_40_H_29_BN_2_O_2_ [M]^+^ 580.2322; found 580.2309 (94%); calcd for C_57_H_48_BN_4_O_2_ [M + DCTB + H]^+^ 831.3870; found 832.3398 (100%).

### Fabrication of the polymer blend samples

The polymer samples were dissolved in toluene to give a 5 wt% solution. Subsequently, the polymer solutions were mixed with a solution of (*R*)-**5** (0.01 M) to give blends in the desired composition with 2 wt% content of (*R*)-**5**. This mixture (300 μL) was spin-coated (1 min, 800 rounds per min) onto a glass substrate, which was allowed to dry for 24 h under ambient conditions.

## Supplementary Material

Supplementary information

Crystal structure data
