# Supplementary material for: A colour-tunable chiral AIEgen: reversible coordination, enantiomer discrimination and morphology visualization
Source: Chem Sci. 2016 Jun 2;7(9):6106–14. doi: 10.1039/c6sc01614f (PMC6024173; doi:10.1039/c6sc01614f)
Supplement: Supplementary file 1 [file SC-007-C6SC01614F-s001.pdf]

Electronic Supplementary Material (ESI) for Chemical Science.
This journal is © The Royal Society of Chemistry 2016

Supplementary information for

A colour-tunable chiral AIEgen: reversible coordination, enantiomer discrimination and morphology visualization

By Jesse Roose, Anakin Chun Sing Leung, Jia Wang, Qian Peng, Herman H. Y. Sung, Ian Duncan Williams and Ben Zhong Tang

Contents

1. Analytical data 2

2. Calculated HOMO and LUMO energies 8

3. UV-vis spectra of the enantiomer discrimination studies 10

4. X-ray crystal data 11

5. References 17

1. Analytical data

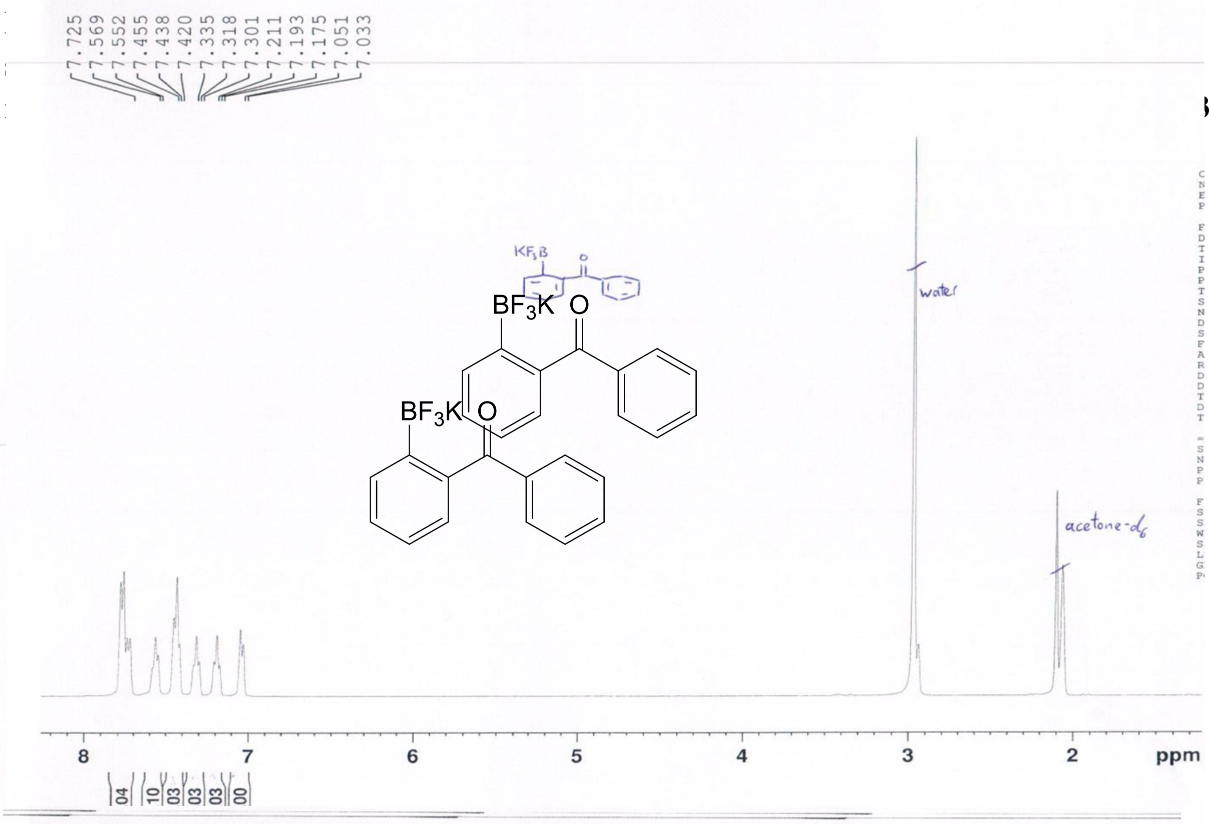

B.

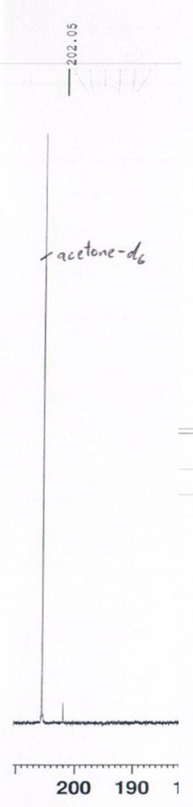

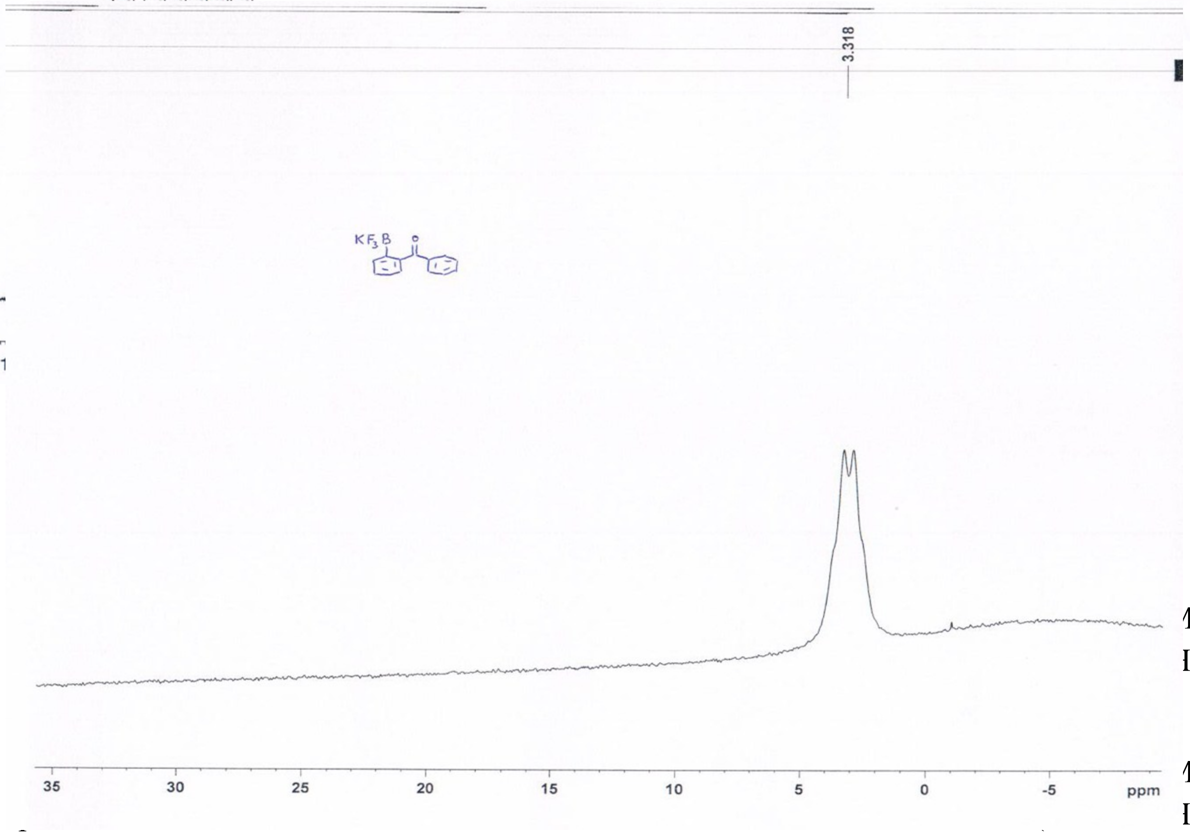

MR
Hz)

MR
Hz)

d_6-acetone of **4**.

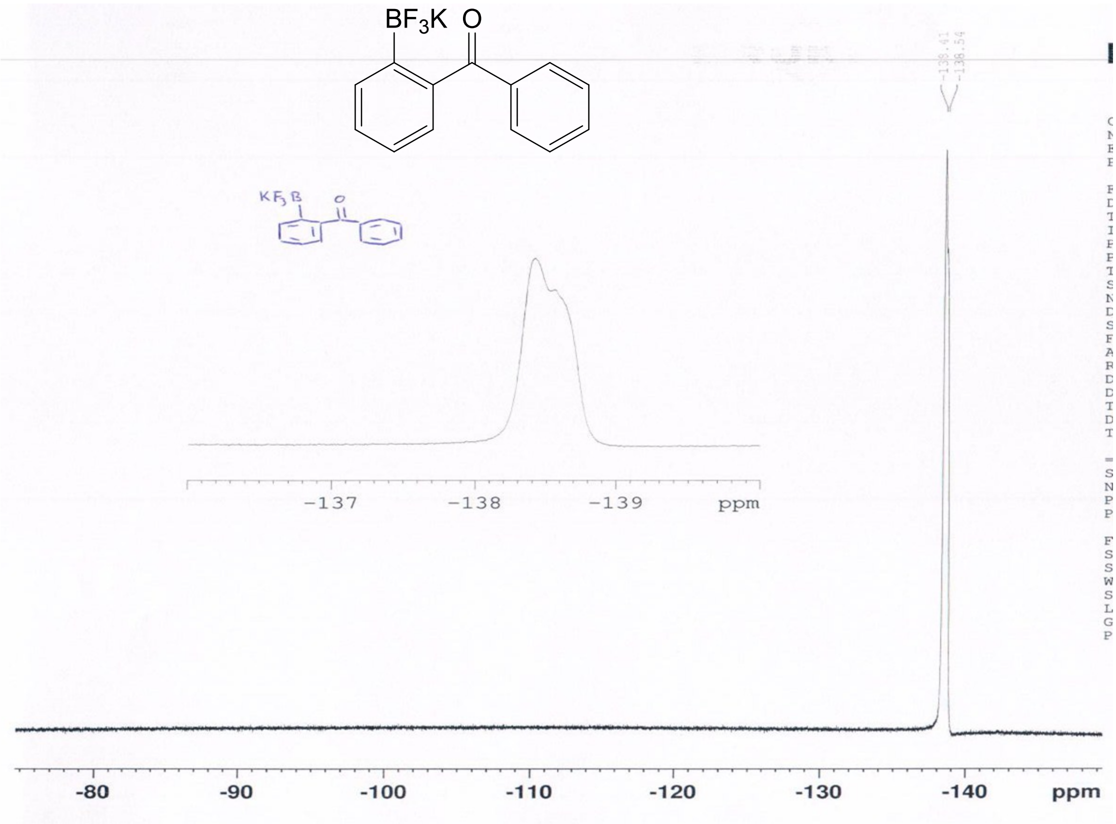

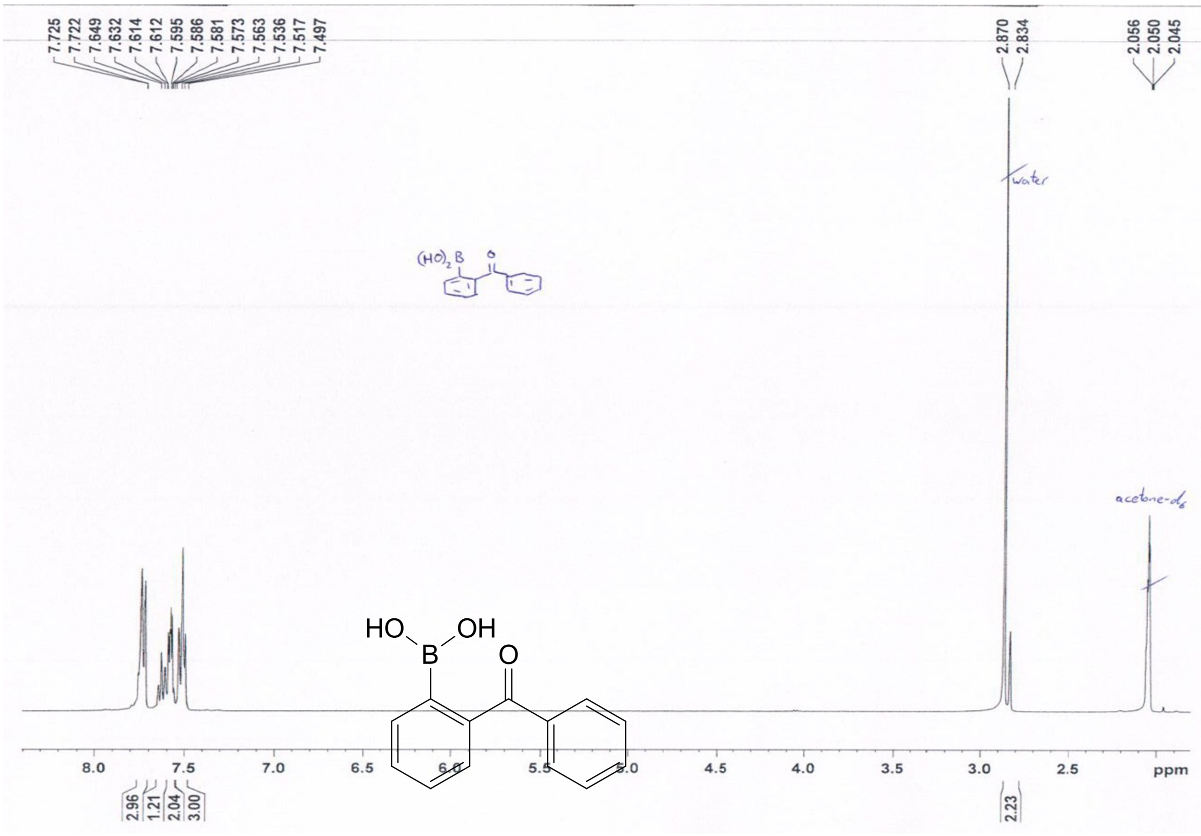

R
z)

R
n

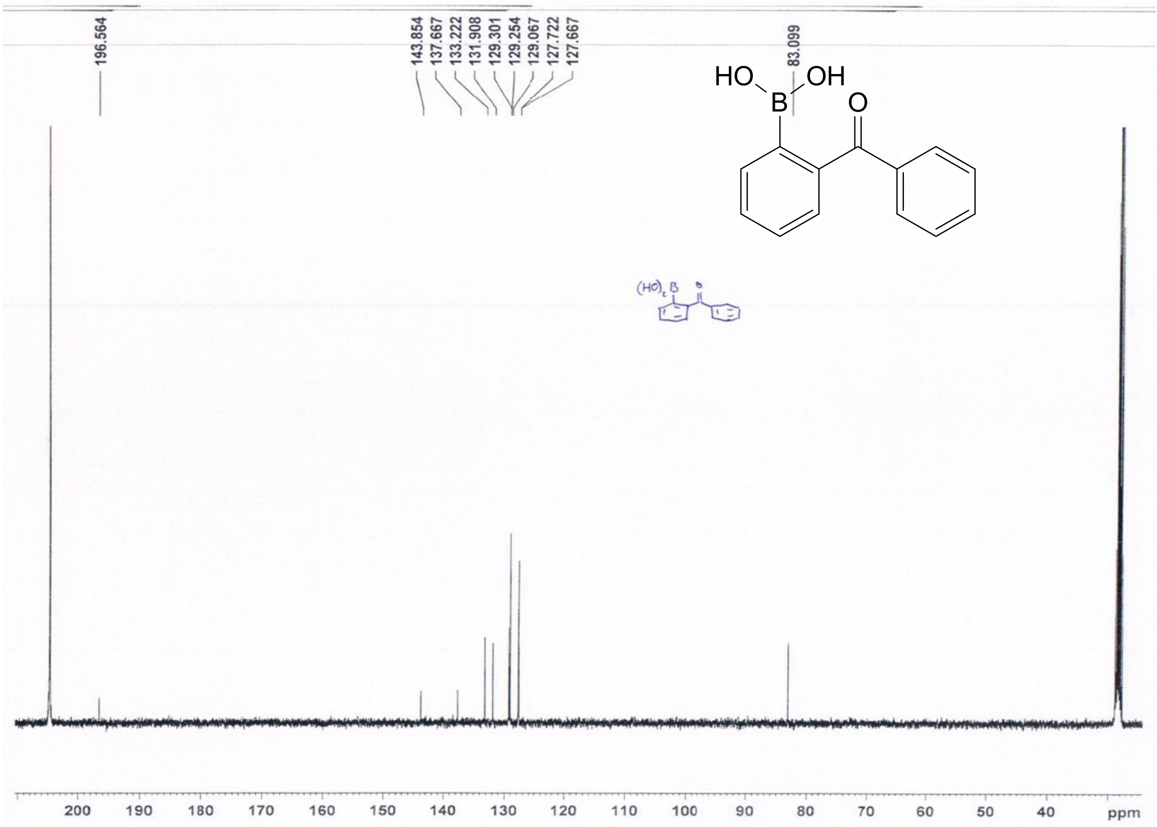

Figure S6. ^{13}C NMR spectrum (100 MHz) d_6-acetone of **4**.

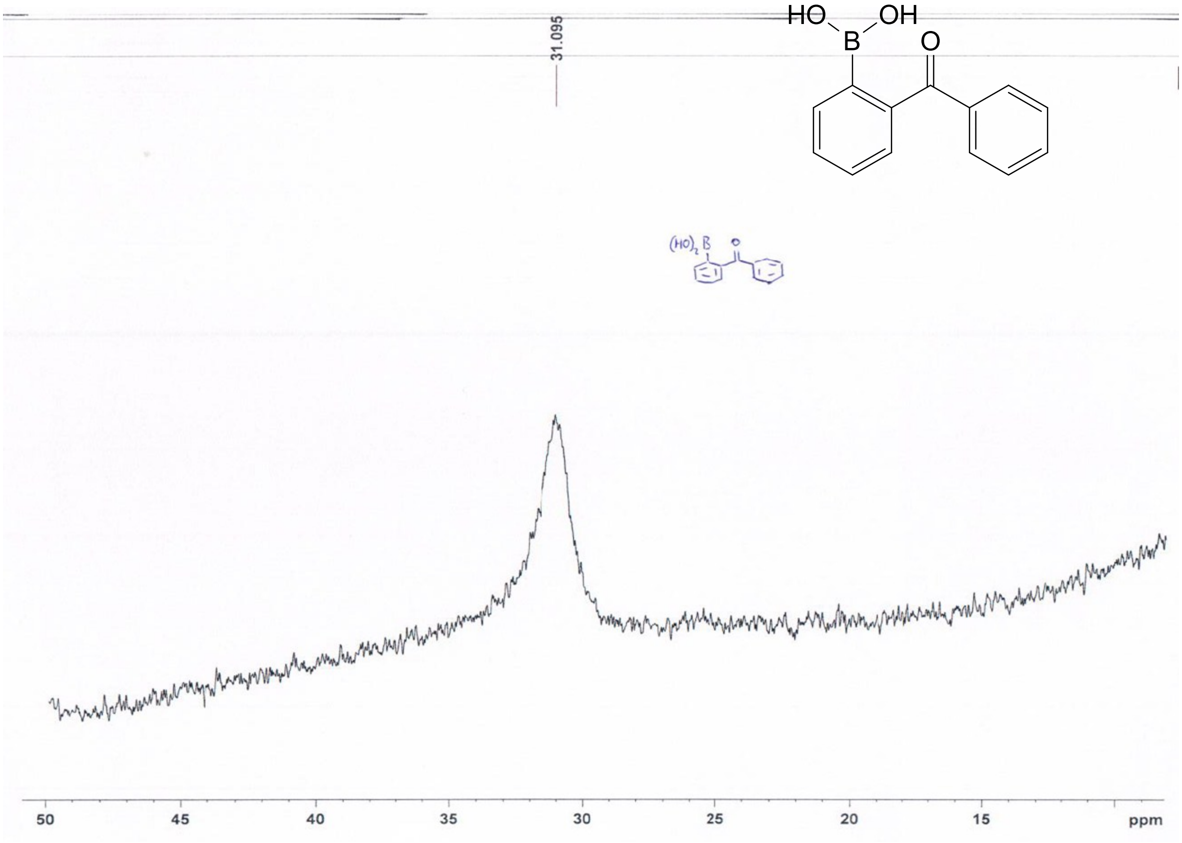
31.095
HO
OH
B
O
(HO)2B
50
45
40
35
30
25
20
15
ppm

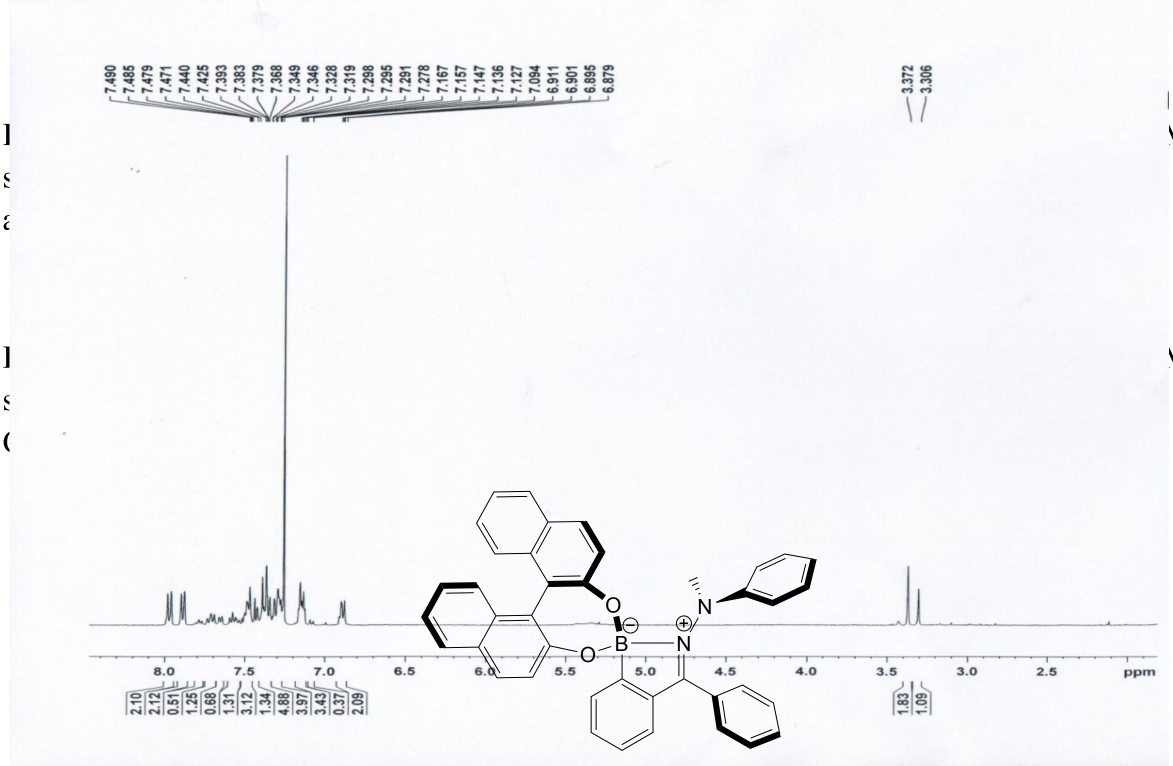
7.490
7.485
7.479
7.471
7.440
7.425
7.393
7.383
7.379
7.368
7.349
7.346
7.328
7.319
7.298
7.295
7.291
7.278
7.167
7.157
7.147
7.136
7.127
7.094
6.911
6.901
6.895
6.879
3.372
3.306
8.0
7.5
7.0
6.5
6.0
5.5
5.0
4.5
4.0
3.5
3.0
2.5
ppm
2.10
2.12
0.51
1.25
0.68
1.31
3.12
1.34
4.88
3.97
3.43
0.37
2.09
1.83
1.09
O
B
N
N

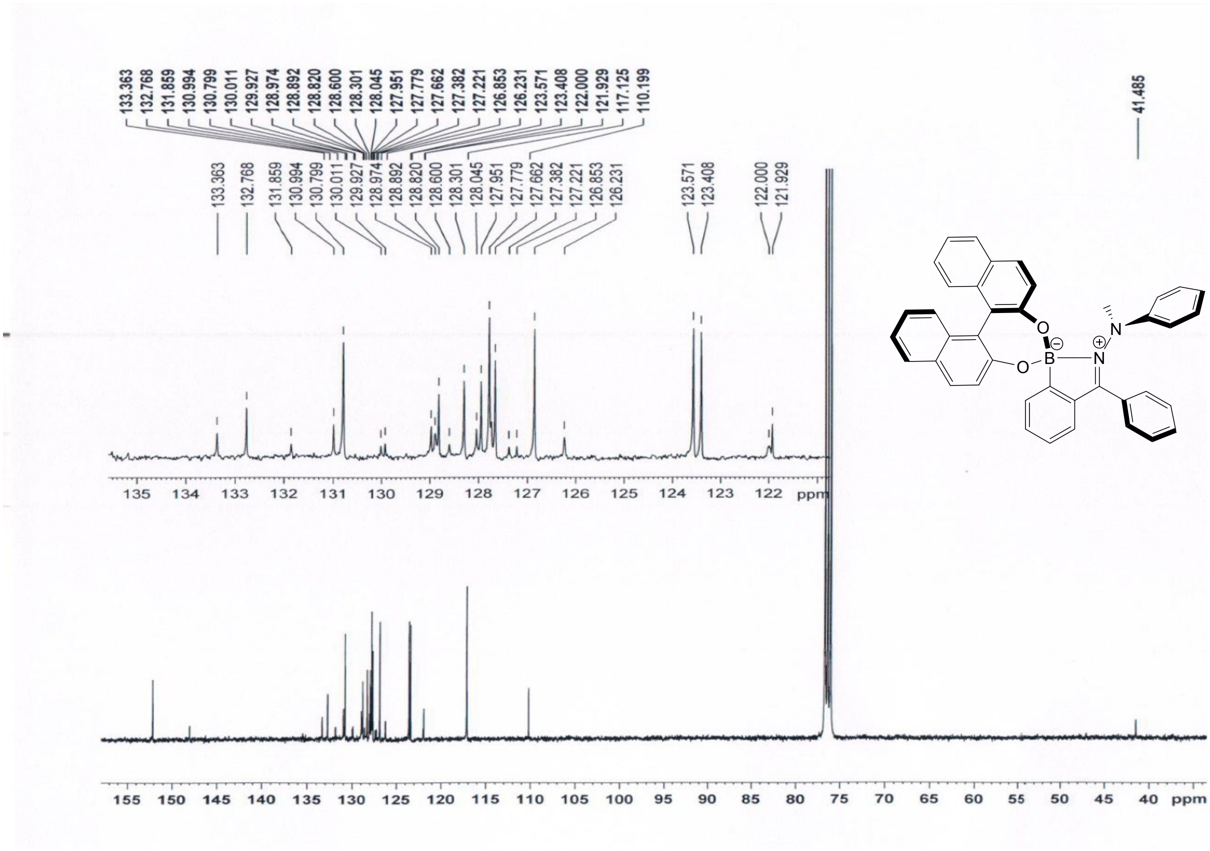

Figure S9. ^{13}C NMR spectrum (100 MHz) in $CDCl_3$ of (*R*)-**5**.

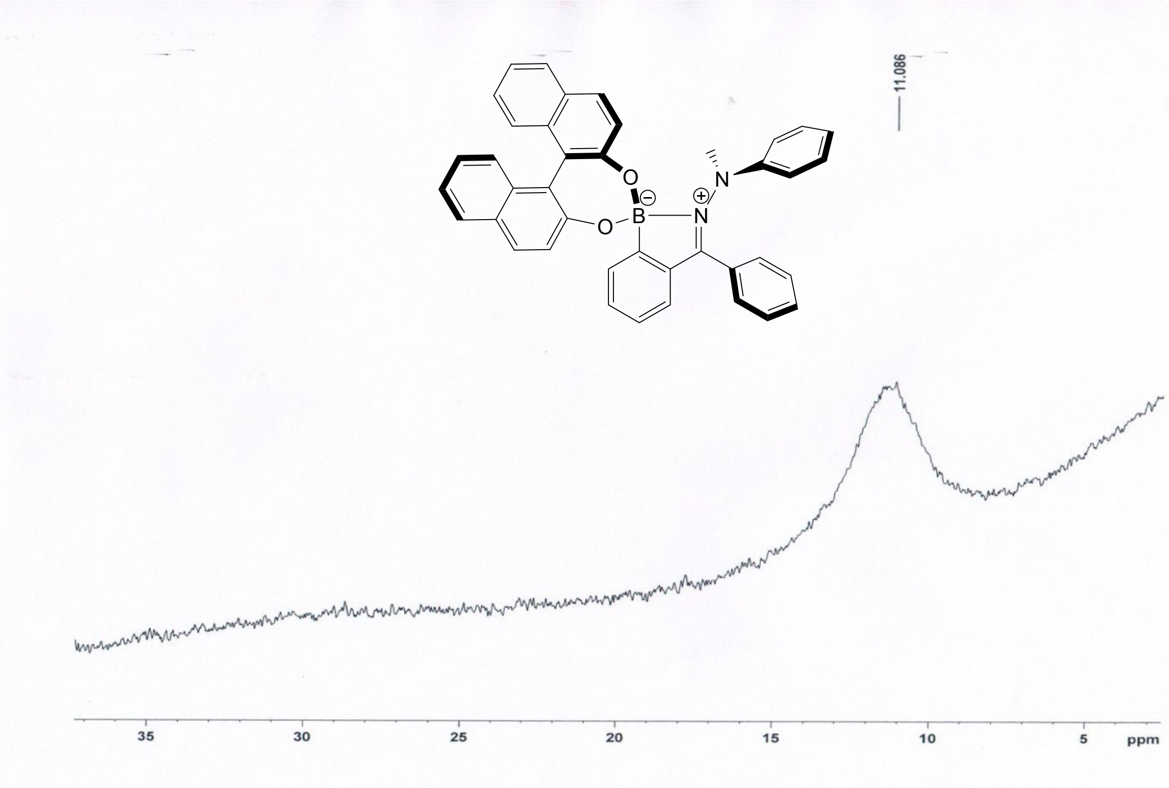

Figure S10. ^{11}B NMR spectrum (128 MHz) in $CDCl_3$ of (*R*)-**5**.

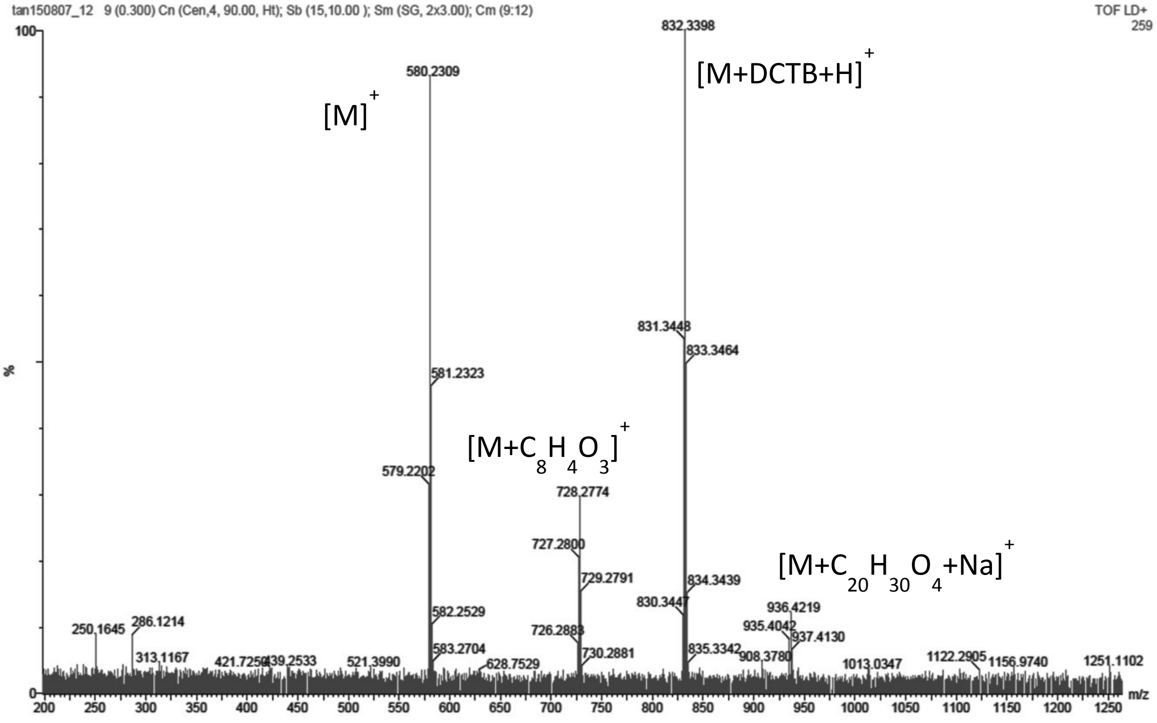

Figure S11. MALDI-ToF-MS with DCTB as matrix of (*R*)-**5**. $C_8H_4O_3$ = phthalic anhydride, DCTB = *trans*-2-[3-(4-*tert*-butylphenyl)-2-methyl-2-propenylidene]malononitrile, $C_{20}H_{30}O_4$ = dihexyl phthalate. The phthalic acid derivatives presumably stemmed from residual PVC-tube softener.

2. Calculated HOMO and LUMO energies

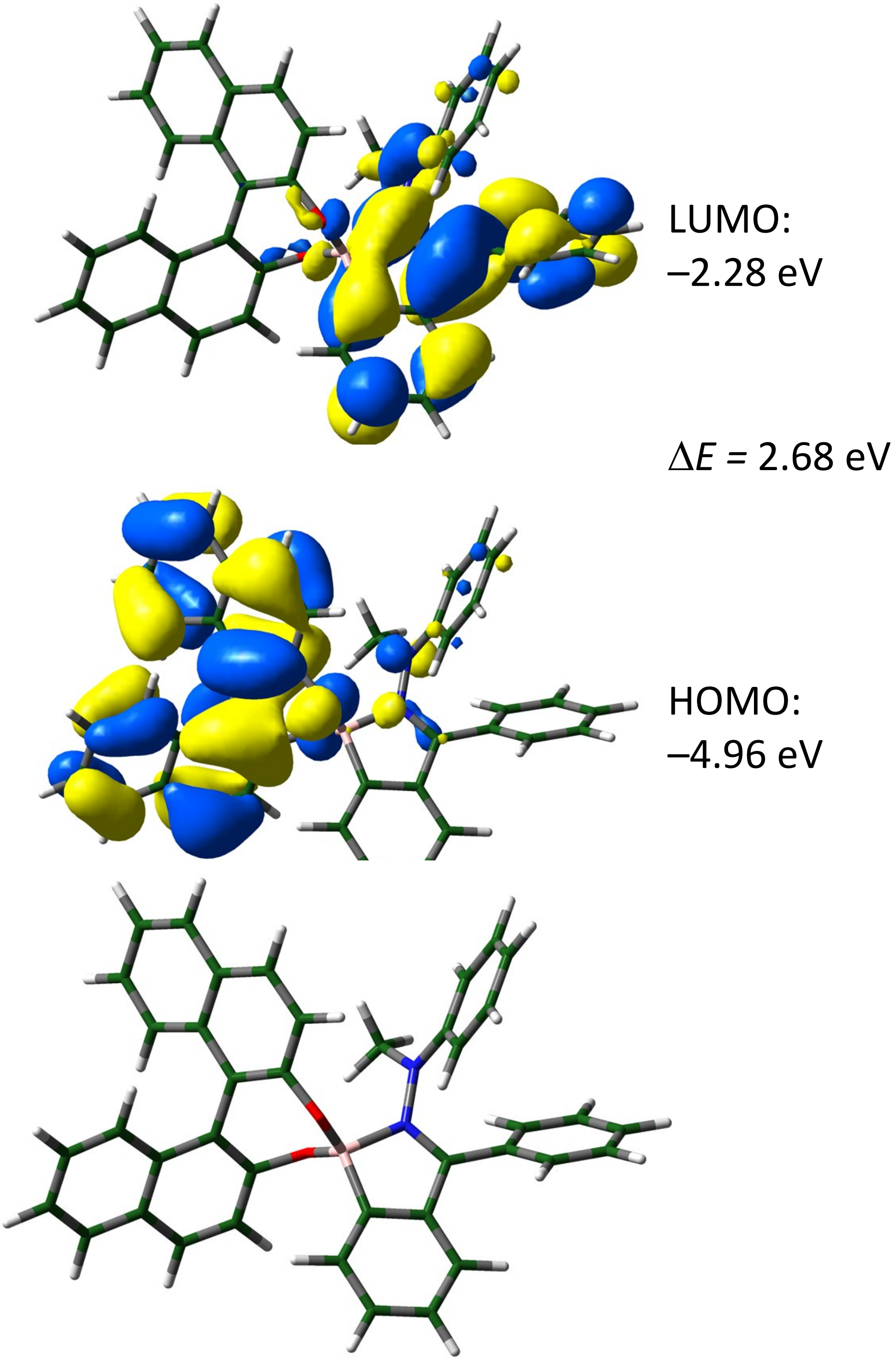

(R)-**5**

Figure S12. Molecular structure obatined from the X-ray crystal analysis and the calculated HOMO/LUMO energies (Gaussian,[1] DFT, B3LYP/6-31G(d)).

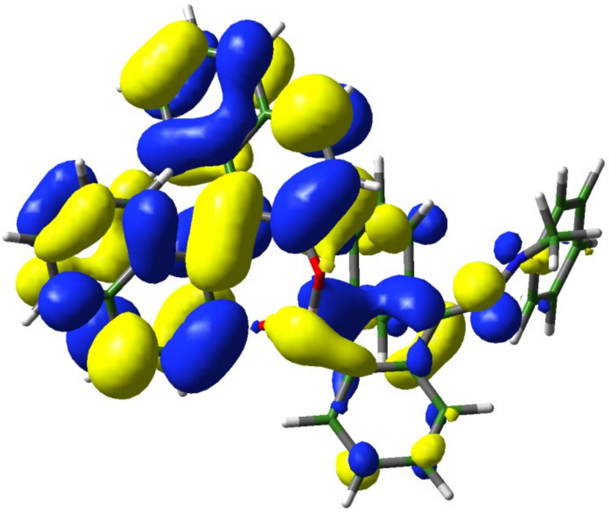

LUMO:
–1.39 eV

ΔE = 3.53 eV

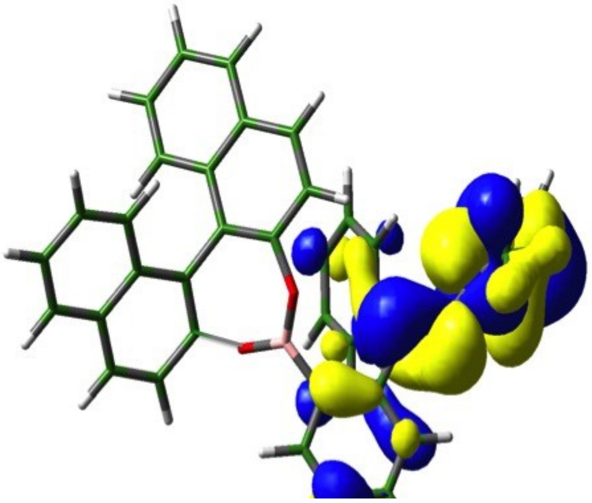

HOMO:
–4.92 eV

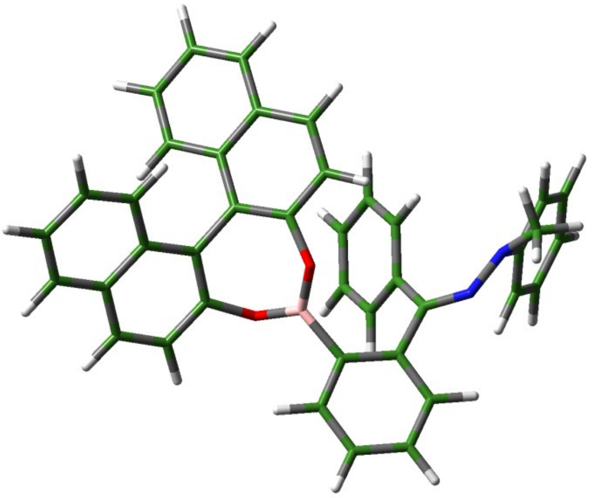

(*R*)-**5** open form

Figure S13. Molecular structure of the open form of (*R*)-**5** and the calculated HOMO/LUMO energies (Gaussian,[1] DFT, B3LYP/6-31G(d)).

3. UV-vis spectra of the enantiomer discrimination studies

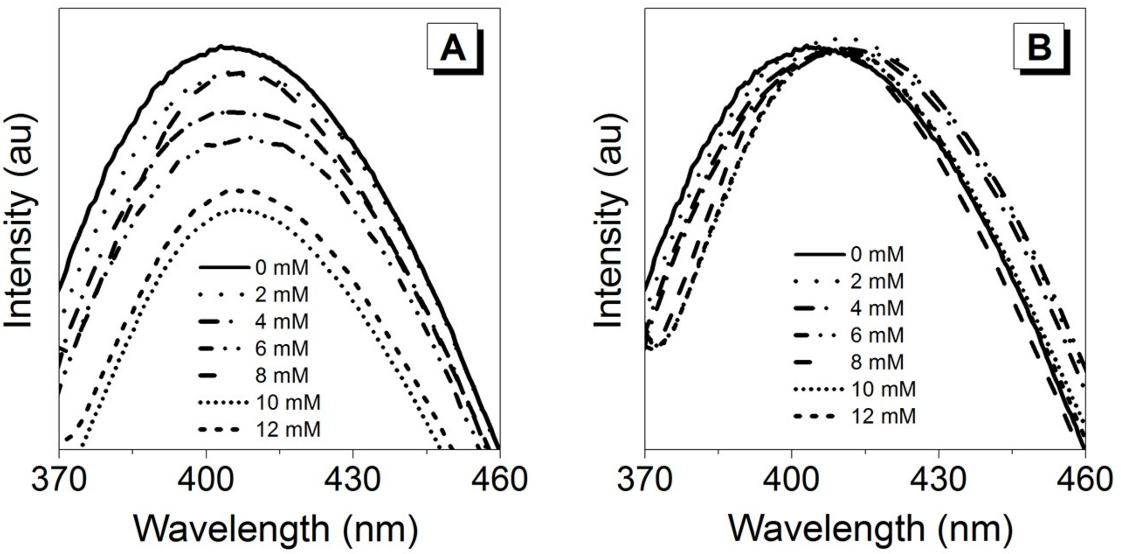

Figure S14. Detailed analysis of the quenching of the absorption band of (*R*)-**5** (10 μM in DCE) at 400 nm as a function of different concentrations (indicated in the graph) of (+)-menthol (A) and (–)-menthol (B).

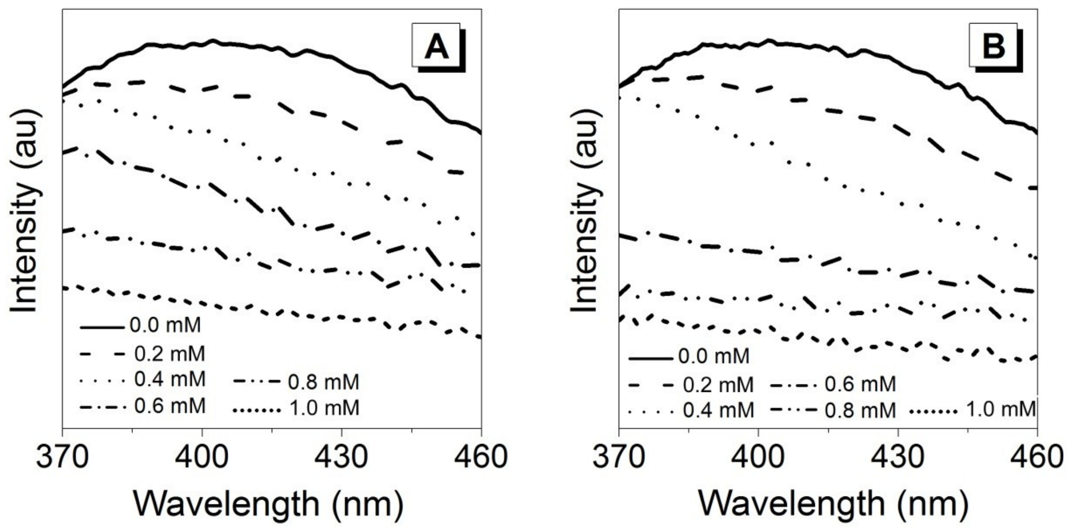

Figure S15. Detailed analysis of the quenching of the absorption band of (*R*)-**5** (10 μM in DCE) at 400 nm as a function of different concentrations (indicated in the graph) of (*R*)-benzylmethylamine (A) and (*S*)-benzylmethylamine (B).

4. X-ray crystal data

A suitable crystal of $C_{40}H_{29}BN_2O_2$, (*R*)-**5** was selected and mounted on a SuperNova, Dual, Cu at zero, Atlas diffractometer. The crystal was kept at 99.99(10) K during data collection. Using Olex2,[2] the structure was solved with the ShelXS[3] structure solution program using Direct Methods and refined with the ShelXL[3] refinement package using Least Squares minimisation.

The crystals were obtained by a vapour diffusion method using 1,2-dichloroethane/hexane.

Table 1 Crystal data of (*R*)-5.

Empirical formula	$C_{40}H_{29}BN_2O_2$
Formula weight	580.46
Temperature/K	99.99(10)
Crystal system	orthorhombic
Space group	$P2_12_12_1$
a/Å	10.13364(19)
b/Å	14.6711(2)
c/Å	19.7677(4)
α/°	90
β/°	90
γ/°	90
Volume/Å^3	2938.89(9)
Z	4
ρ_{calc}g/cm^3	1.312
μ/mm^{-1}	0.628
F(000)	1216.0
Crystal size/mm^3	0.2 × 0.18 × 0.05
Radiation	CuKα (λ = 1.54184)
2Θ range for data collection/°	8.946 to 134.948
Index ranges	$-12 \leq h \leq 11$, $-17 \leq k \leq 13$, $-21 \leq l \leq 23$
Reflections collected	16496
Independent reflections	5230 [R_{int} = 0.0387, R_{sigma} = 0.0386]
Data/restraints/parameters	5230/0/407
Completeness to theta = 66.5°	99.4%
Goodness-of-fit on F^2	1.002
Final R indexes [I>=2σ (I)]	R_1 = 0.0330, wR_2 = 0.0791
Final R indexes [all data]	R_1 = 0.0404, wR_2 = 0.0824
Largest diff. peak/hole / e Å^{-3}	0.27/-0.14
Flack parameter	0.01(15)

Table 2 Bond Lengths for (*R*)-5.

Atom	Atom	Length/Å	Atom	Atom	Length/Å
O1	B1	1.453(3)	C22	C32	1.491(3)
O1	C21	1.364(3)	C23	C24	1.416(3)
O2	B1	1.447(3)	C23	C28	1.430(3)
O2	C31	1.360(3)	C24	C25	1.369(3)
N1	N2	1.412(3)	C25	C26	1.417(4)
N1	B1	1.663(3)	C26	C27	1.364(4)
N1	C1	1.300(3)	C27	C28	1.421(3)
N2	C10	1.466(3)	C28	C29	1.412(4)
N2	C11	1.402(3)	C29	C30	1.363(4)
B1	C3	1.611(3)	C31	C32	1.388(3)
C1	C2	1.467(3)	C31	C40	1.415(3)
C1	C41	1.473(3)	C32	C33	1.431(3)
C2	C3	1.401(3)	C33	C34	1.424(3)
C2	C7	1.396(3)	C33	C38	1.431(3)
C3	C4	1.386(3)	C34	C35	1.376(3)
C4	C5	1.398(4)	C35	C36	1.403(4)
C5	C6	1.392(4)	C36	C37	1.366(4)
C6	C7	1.377(4)	C37	C38	1.410(3)
C11	C12	1.407(3)	C38	C39	1.416(3)
C11	C16	1.390(4)	C39	C40	1.365(3)
C12	C13	1.383(4)	C41	C42	1.397(3)
C13	C14	1.382(4)	C41	C46	1.385(4)
C14	C15	1.385(4)	C42	C43	1.382(3)
C15	C16	1.386(4)	C43	C44	1.377(4)
C21	C22	1.385(3)	C44	C45	1.379(4)
C21	C30	1.410(3)	C45	C46	1.387(3)
C22	C23	1.432(3)			

Table 3 Bond Angles for (*R*)-5.

Atom	Atom	Atom	Angle/°	Atom	Atom	Atom	Angle/°
C21	O1	B1	113.47(17)	C23	C22	C32	122.44(19)
C31	O2	B1	120.66(18)	C24	C23	C22	123.0(2)
N2	N1	B1	127.49(17)	C24	C23	C28	117.8(2)
C1	N1	N2	120.2(2)	C28	C23	C22	119.2(2)
C1	N1	B1	112.08(19)	C25	C24	C23	121.6(2)
N1	N2	C10	112.62(19)	C24	C25	C26	120.2(2)
C11	N2	N1	118.47(19)	C27	C26	C25	120.0(2)
C11	N2	C10	119.5(2)	C26	C27	C28	120.9(2)
O1	B1	N1	105.35(18)	C27	C28	C23	119.4(2)
O1	B1	C3	116.6(2)	C29	C28	C23	119.1(2)
O2	B1	O1	114.59(18)	C29	C28	C27	121.6(2)
O2	B1	N1	109.27(18)	C30	C29	C28	121.0(2)
O2	B1	C3	112.6(2)	C29	C30	C21	120.0(2)
C3	B1	N1	96.19(17)	O2	C31	C32	121.5(2)
N1	C1	C2	111.1(2)	O2	C31	C40	117.2(2)
N1	C1	C41	125.9(2)	C32	C31	C40	121.2(2)
C2	C1	C41	123.0(2)	C31	C32	C22	119.5(2)
C3	C2	C1	110.8(2)	C31	C32	C33	118.4(2)
C7	C2	C1	126.6(2)	C33	C32	C22	121.9(2)
C7	C2	C3	122.5(2)	C32	C33	C38	119.9(2)
C2	C3	B1	109.6(2)	C34	C33	C32	123.3(2)
C4	C3	B1	132.6(2)	C34	C33	C38	116.8(2)
C4	C3	C2	117.7(2)	C35	C34	C33	121.3(2)
C3	C4	C5	120.4(2)	C34	C35	C36	120.8(2)
C6	C5	C4	120.7(2)	C37	C36	C35	119.6(2)
C7	C6	C5	120.1(2)	C36	C37	C38	121.1(2)
C6	C7	C2	118.6(2)	C37	C38	C33	120.1(2)
N2	C11	C12	118.5(2)	C37	C38	C39	121.0(2)
C16	C11	N2	122.7(2)	C39	C38	C33	118.8(2)
C16	C11	C12	118.8(2)	C40	C39	C38	120.6(2)
C13	C12	C11	120.0(2)	C39	C40	C31	120.7(2)
C14	C13	C12	121.3(3)	C42	C41	C1	117.1(2)
C13	C14	C15	118.5(3)	C46	C41	C1	122.7(2)
C14	C15	C16	121.4(2)	C46	C41	C42	120.2(2)
C15	C16	C11	120.0(2)	C43	C42	C41	119.9(2)
O1	C21	C22	120.4(2)	C44	C43	C42	119.7(2)
O1	C21	C30	118.09(19)	C43	C44	C45	120.5(2)
C22	C21	C30	121.4(2)	C44	C45	C46	120.6(2)
C21	C22	C23	118.5(2)	C41	C46	C45	119.1(2)
C21	C22	C32	119.0(2)				

Table 4 Torsion Angles for (*R*)-5.

A	B	C	D	Angle/°	A	B	C	D	Angle/°
O1	B1	C3	C2	111.9(2)	C21	O1	B1	O2	-59.4(3)
O1	B1	C3	C4	-64.2(3)	C21	O1	B1	N1	-179.49(16)
O1	C21	C22	C23	-173.19(19)	C21	O1	B1	C3	75.3(2)
O1	C21	C22	C32	7.7(3)	C21	C22	C23	C24	168.9(2)
O1	C21	C30	C29	179.9(2)	C21	C22	C23	C28	-9.2(3)
O2	B1	C3	C2	-112.6(2)	C21	C22	C32	C31	-49.7(3)
O2	B1	C3	C4	71.3(3)	C21	C22	C32	C33	125.8(2)
O2	C31	C32	C22	-2.9(3)	C22	C21	C30	C29	-2.7(3)
O2	C31	C32	C33	-178.5(2)	C22	C23	C24	C25	-179.8(2)
O2	C31	C40	C39	-177.4(2)	C22	C23	C28	C27	-178.6(2)
N1	N2	C11	C12	-165.8(2)	C22	C23	C28	C29	2.5(3)
N1	N2	C11	C16	14.8(3)	C22	C32	C33	C34	-4.3(3)
N1	B1	C3	C2	1.3(2)	C22	C32	C33	C38	177.4(2)
N1	B1	C3	C4	-174.8(2)	C23	C22	C32	C31	131.2(2)
N1	C1	C2	C3	-4.9(3)	C23	C22	C32	C33	-53.3(3)
N1	C1	C2	C7	171.3(2)	C23	C24	C25	C26	-1.3(4)
N1	C1	C41	C42	125.9(3)	C23	C28	C29	C30	4.4(3)
N1	C1	C41	C46	-54.8(4)	C24	C23	C28	C27	3.3(3)
N2	N1	B1	O1	61.4(3)	C24	C23	C28	C29	-175.7(2)
N2	N1	B1	O2	-62.2(3)	C24	C25	C26	C27	2.7(4)
N2	N1	B1	C3	-178.8(2)	C25	C26	C27	C28	-1.0(4)
N2	N1	C1	C2	-179.32(19)	C26	C27	C28	C23	-2.0(4)
N2	N1	C1	C41	2.9(4)	C26	C27	C28	C29	177.0(2)
N2	C11	C12	C13	-179.5(3)	C27	C28	C29	C30	-174.5(2)
N2	C11	C16	C15	179.5(2)	C28	C23	C24	C25	-1.7(3)
B1	O1	C21	C22	68.1(3)	C28	C29	C30	C21	-4.5(3)
B1	O1	C21	C30	-114.4(2)	C30	C21	C22	C23	9.4(3)
B1	O2	C31	C32	60.8(3)	C30	C21	C22	C32	-169.7(2)
B1	O2	C31	C40	-122.6(2)	C31	O2	B1	O1	-23.9(3)
B1	N1	N2	C10	-54.4(3)	C31	O2	B1	N1	94.1(2)
B1	N1	N2	C11	92.1(3)	C31	O2	B1	C3	-160.3(2)
B1	N1	C1	C2	5.8(3)	C31	C32	C33	C34	171.2(2)
B1	N1	C1	C41	-171.9(2)	C31	C32	C33	C38	-7.0(3)
B1	C3	C4	C5	175.4(2)	C32	C22	C23	C24	-12.0(3)
C1	N1	N2	C10	131.6(2)	C32	C22	C23	C28	169.9(2)
C1	N1	N2	C11	-81.9(3)	C32	C31	C40	C39	-0.8(4)
C1	N1	B1	O1	-124.2(2)	C32	C33	C34	C35	179.0(2)
C1	N1	B1	O2	112.2(2)	C32	C33	C38	C37	-176.3(2)
C1	N1	B1	C3	-4.4(2)	C32	C33	C38	C39	4.9(3)
C1	C2	C3	B1	1.7(3)	C33	C34	C35	C36	-1.6(4)
C1	C2	C3	C4	178.5(2)	C33	C38	C39	C40	-0.7(3)
C1	C2	C7	C6	-177.6(2)	C34	C33	C38	C37	5.4(3)
C1	C41	C42	C43	179.0(2)	C34	C33	C38	C39	-173.4(2)
C1	C41	C46	C45	-177.5(2)	C34	C35	C36	C37	3.4(4)

C2	C1	C41	C42	-51.6(3)	C35	C36	C37	C38	-0.6(4)
C2	C1	C41	C46	127.7(3)	C36	C37	C38	C33	-3.8(4)
C2	C3	C4	C5	-0.4(3)	C36	C37	C38	C39	175.0(2)
C3	C2	C7	C6	-1.8(4)	C37	C38	C39	C40	-179.5(2)
C3	C4	C5	C6	-1.4(4)	C38	C33	C34	C35	-2.7(3)
C4	C5	C6	C7	1.7(4)	C38	C39	C40	C31	-1.4(4)
C5	C6	C7	C2	-0.2(4)	C40	C31	C32	C22	-179.4(2)
C7	C2	C3	B1	-174.7(2)	C40	C31	C32	C33	5.0(3)
C7	C2	C3	C4	2.0(3)	C41	C1	C2	C3	172.9(2)
C10	N2	C11	C12	-21.7(3)	C41	C1	C2	C7	-10.9(4)
C10	N2	C11	C16	158.9(2)	C41	C42	C43	C44	-2.1(4)
C11	C12	C13	C14	0.3(5)	C42	C41	C46	C45	1.7(4)
C12	C11	C16	C15	0.1(3)	C42	C43	C44	C45	2.9(4)
C12	C13	C14	C15	-0.4(5)	C43	C44	C45	C46	-1.5(4)
C13	C14	C15	C16	0.4(4)	C44	C45	C46	C41	-0.9(4)
C14	C15	C16	C11	-0.2(4)	C46	C41	C42	C43	-0.2(4)
C16	C11	C12	C13	-0.2(4)					

References

[1] Frisch, M. J.; Trucks, G. W.; Schlegel, H. B.; Scuseria, G. E.; Robb, M. A.; Cheeseman, J. R.; Scalmani, G.; Barone, V.; Mennucci, B.; Petersson, G. A.; Nakatsuji, H.; Caricato, M.; Li, X.; Hratchian, H. P.; Izmaylov, A. F.; Bloino, J.; Zheng, G.; Sonnenberg, J. L.; Hada, M.; Ehara, M.; Toyota, K.; Fukuda, R.; Hasegawa, J.; Ishida, M.; Nakajima, T.; Honda, Y.; Kitao, O.; Nakai, H.; Vreven, T.; Montgomery Jr., J. A.; Peralta, J. E.; Ogliaro, F.; Bearpark, M. J.; Heyd, J.; Brothers, E. N.; Kudin, K. N.; Staroverov, V. N.; Kobayashi, R.; Normand, J.; Raghavachari, K.; Rendell, A. P.; Burant, J. C.; Iyengar, S. S.; Tomasi, J.; Cossi, M.; Rega, N.; Millam, N. J.; Klene, M.; Knox, J. E.; Cross, J. B.; Bakken, V.; Adamo, C.; Jaramillo, J.; Gomperts, R.; Stratmann, R. E.; Yazyev, O.; Austin, A. J.; Cammi, R.; Pomelli, C.; Ochterski, J. W.; Martin, R. L.; Morokuma, K.; Zakrzewski, V. G.; Voth, G. A.; Salvador, P.; Dannenberg, J. J.; Dapprich, S.; Daniels, A. D.; Farkas, Ö.; Foresman, J. B.; Ortiz, J. V.; Cioslowski, J.; Fox, D. J.: Gaussian 09. Gaussian, Inc.: Wallingford, CT, USA, 2009.

[2] O. V. Dolomanov, L. J. Bourhis, R. J. Gildea, J. A. K. Howard, H. Puschmann, *J. Appl. Cryst.* 2009, **42**, 339.

[3] G. M. Sheldrick, *Acta Cryst.* 2008, **A64**, 112.
